# Multiple patho-phenotyping and molecular analysis to characterize wide-spectrum durable leaf rust resistance in wheat collections from India

**DOI:** 10.3389/fmicb.2025.1596282

**Published:** 2025-07-03

**Authors:** Ram Mohan, Vaibhav Kumar Singh, K. K. Chetan, Lingareddy Usha Rani, Koshal K. Sameriya, Subodh Kumar, Naresh Kumar Bainsla, Govindasamy Senthilraja, Mahender Singh Saharan

**Affiliations:** ^1^Wheat Pathology Laboratory, Division of Plant Pathology, ICAR-Indian Agricultural Research Institute, New Delhi, India; ^2^ICAR-Indian Institute of Wheat and Barley Research, Shimla, Himachal Pradesh, India; ^3^Division of Genetics, ICAR-Indian Agricultural Research Institute, New Delhi, India; ^4^Department of Plant Pathology, Centre for Plant Protection Studies, Tamil Nadu Agricultural University, Coimbatore, India

**Keywords:** wheat, *Puccinia triticina*, leaf rust, gene postulation, *Lr* genes, epidemiological parameters, adult plant resistance, molecular markers

## Abstract

Wheat leaf rust, caused by *Puccinia triticina* (*Pt*), is a globally prevalent fungal disease that causes significant economic loss. Cultivar resistance remains a cornerstone of the management of this pathogen. This study evaluated 86 Indian wheat (*Triticum aestivum L*.) genotypes to characterize leaf rust resistance (*Lr*) genes, assess adult plant resistance (APR) under field conditions, and validate resistance using molecular marker analysis. Seedling resistance tests against 14 *Pt* pathotypes identified nine key *Lr* genes (*Lr*1, *Lr*3, *Lr*10, *Lr*14a, *Lr*16, *Lr*23, *Lr*24, *Lr*26, and *Lr*34) in 26 genotypes, either alone or in combination with other resistance genes. Field evaluations across two consecutive rabi seasons (2020–21 and 2021–22) revealed quantitative, partial, non-race-specific, slow-rusting APR in over 64 genotypes. These genotypes, which are susceptible to prevalent *Pt* pathotypes at the seedling stage, demonstrated that APR is mediated by minor-effect genes. Epidemiological parameters (final disease severity, coefficient of infection, relative area under the disease progression curve, and infection rate) showed strong positive correlations, validating their utility for quantifying slow-rusting resistance. Molecular analysis detected *Lr*34 in 33 genotypes, followed by *Lr*10 (24 genotypes), and *Lr*24 (16 genotypes), confirming their role in conferring resistance. Genotypes that combine seedling and APR resistance, particularly those harboring *Lr*34, *Lr*10, or *Lr*24, offer valuable genetic resources for breeding programs. Their integration into wheat improvement initiatives can enhance resistance against evolving *Pt* pathotypes, mitigate yield losses, and contribute to sustainable wheat production. This study underscores the importance of deploying multigenic resistance strategies to counter rapid pathogen evolution and ensure long-term disease management.

## Introduction

Wheat (*Triticum* species) is one of the most important cereal crops globally, and it plays a pivotal role in ensuring food security and sustaining agricultural productivity. As the most widely cultivated food crop, wheat occupies approximately 219.62 million hectares of land and produces an estimated 789.34 million metric tons annually (USDA, 2024). India is the world’s second-largest producer and consumer of wheat after China, driven by significant improvements in productivity and production. According to the Ministry of Agriculture and Farmers Welfare, Government of India, the country is projected to produce a record of 115.5 million metric tons of wheat during 2024–25, based on second-advance estimates of national foodgrain output (PIB, 2024). However, sustaining wheat productivity is essential for meeting the demands of a growing population. By 2050, India will require over 140 million metric tons of wheat to feed its projected population of 1.64 billion people (Bhardwaj et al., 2019; Mottaleb et al., 2023). Achieving this target requires innovative strategies to mitigate biotic stresses that constrain wheat production.

Among biotic stresses, pests and diseases account for approximately 21.9% of the yield losses in wheat (Savary et al., 2019). Fungal pathogens are the primary culprits, with three rust diseases, *viz*., leaf (brown) rust, stripe (yellow) rust, and stem (black) rust, causing the most significant damage, followed by powdery mildew and Karnal bunt (Ul Islam et al., 2023; Singh et al., 2023). Wheat rust has historically caused substantial yield reductions worldwide, including India (Herrera-Foessel et al., 2011; Bhardwaj et al., 2019; Srinivas et al., 2021). The occurrence and severity of rust events depend on various factors, including meteorological conditions, crop growth stages, and host resistance levels, with the potential to reduce yields by up to 100% under severe infestations (Figueroa et al., 2018; Prank et al., 2019).

Among the three rusts, leaf rust, caused by *Puccinia triticina* Eriks (*Pt*), is the most prevalent and widely distributed, affecting 94.4% of the global wheat production (Yan et al., 2021). This pathogen thrives across diverse environments and is ubiquitous in wheat (Winzeler et al., 2000; Meyer et al., 2021). Although less destructive than stem or stripe rust, leaf rust frequently causes higher annual losses owing to its widespread occurrence (Chen et al., 2014). Under favorable conditions, yield losses from leaf rust can exceed 50%, and acute infestations of susceptible varieties may result in total yield losses of 50–70% (Herrera-Foessel et al., 2011; Malysheva et al., 2023). The primary cause of these losses is infection during the flag leaf stage, which is critical for grain filling and physiological processes essential for yield formation (Ordoñez et al., 2010). In India, leaf rust is the most commonly observed wheat disease with a well-documented propensity to spread across wheat fields (Bhardwaj et al., 2019; Srinivas et al., 2021; Figueroa et al., 2018). Recently identified aggressive *Pt* races such as 77–5 (121R63-1), 77–9 (121R60-1), and 104–2 (21R55) have rendered numerous high-yielding wheat cultivars vulnerable, highlighting the urgent need for durable resistance (Bhardwaj et al., 2019; Bhardwaj et al., 2021). Furthermore, the genetic uniformity of many modern cultivars, stemming from their common ancestry, exacerbates their susceptibility to novel pathotypes, thereby increasing the risk of catastrophic outbreaks.

To date, more than a 100 leaf rust resistance (*Lr*) genes and alleles have been identified (Tong et al., 2024). Most confer race-specific resistance at the seedling stage, whereas a few confer adult plant resistance (APR). Only a handful of *Lr* genes, including *Lr34*, *Lr46*, *Lr67*, and *Lr68*, offer non-race-specific or slow-rusting APR, which are more durable and robust than seedling-stage resistance (Herrera-Foessel et al., 2012). The most effective strategy for mitigating leaf rust damage and sustaining wheat productivity is to enhance the host resistance (Mapuranga et al., 2022). Historically, breeding efforts have been focused on qualitative and race-specific resistance. However, the transient nature of the hypersensitive resistance underscores the need for quantitative and durable resistance mechanisms (Maurya et al., 2021). Recent studies have indicated that many wheat cultivars carrying primary *Lr* genes, such as *Lr9*, *Lr19*, and *Lr28*, are no longer effective against contemporary pathotypes. Consequently, identifying novel sources of resistance that confer resilience and long-lasting protection against rust is imperative for wheat scientists worldwide. Current research emphasizes deploying a combined strategy that integrates both major (race-specific/seedling) and minor (non-race-specific/APR) resistance genes, which are considered to be more durable and effective for long-term leaf rust management (Singh et al., 2004; Merrick et al., 2021). Additionally, the availability of gene-linked molecular markers facilitates targeted breeding strategies for rust resistance. Exploration of wheat genetic resources with diverse resistance mechanisms is essential for efficient leaf rust control. Phenotyping and screening of genotypes for resistance remain the most effective methods for identifying novel sources suitable for cultivation in leaf rust-prone regions.

**Figure 1:**
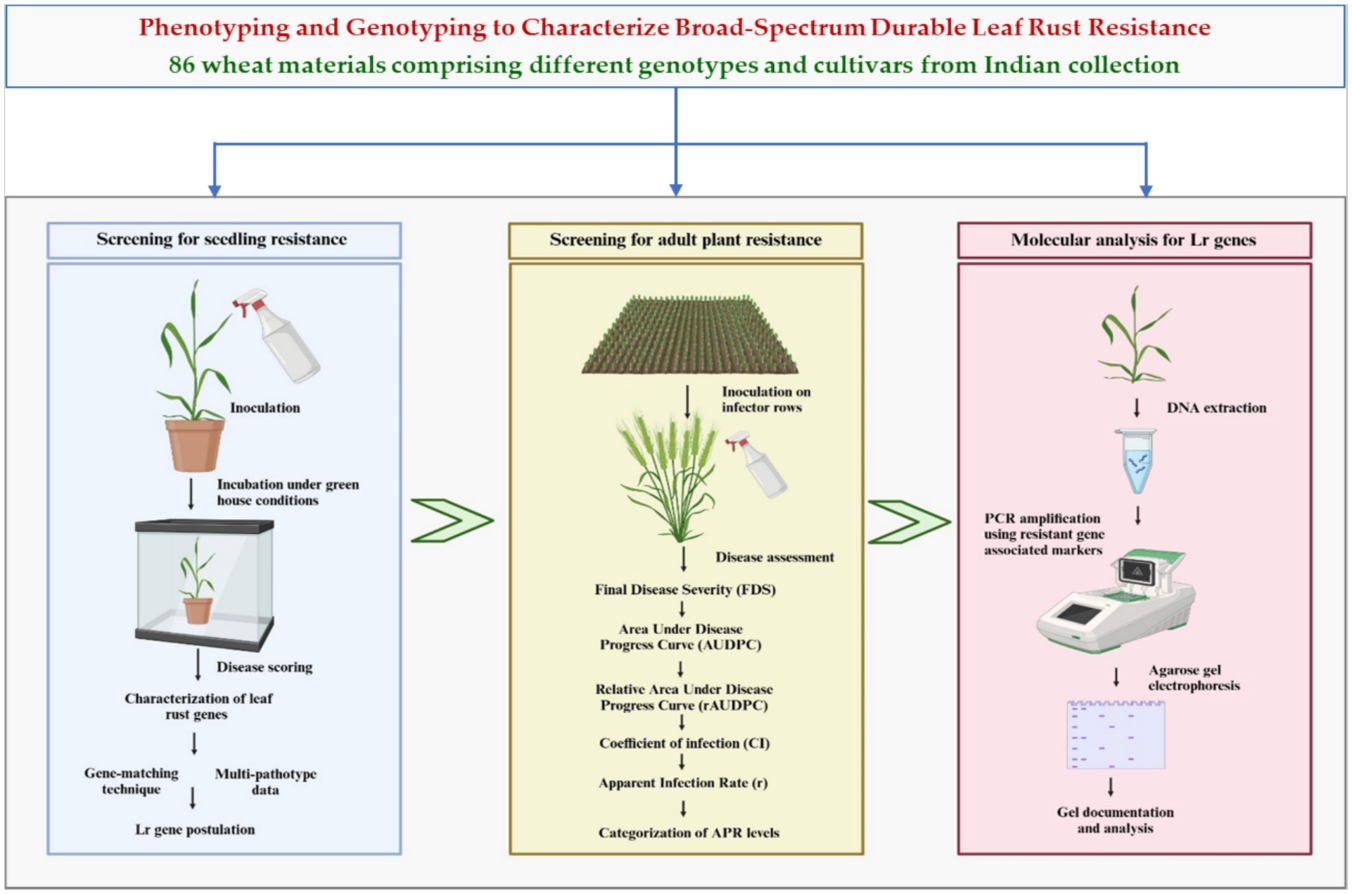
Flow diagram describing the procedure adopted in the study.

This study aimed to evaluate the resistance of a collection of Indian wheat genotypes and cultivars to leaf rust at both seedling and adult plant stages. Resistance genes were confirmed using molecular markers linked to known *Lr* genes. A schematic representation of the experimental workflow is shown in Figure 1. The overarching objective of this study was to identify potential resistance sources and combinations of strong genes for integration into wheat hybridization programs focused on enhancing rust resistance. By addressing these objectives, the present study contributes to the development of resilient wheat varieties that can sustain productivity in the face of evolving biotic threats.

## Materials and methods

### Plant materials

A total of 86 wheat samples, including diverse genotypes and cultivars sourced from indigenous collections representing the major wheat-growing zones of India, were selected for this study. Wheat seeds were obtained from the Division of Genetics of the ICAR-Indian Agricultural Research Institute (IARI), New Delhi. Detailed information regarding the pedigree and origin of wheat resources is provided in Supplementary Table S1. To ensure an accurate evaluation, two highly susceptible wheat cultivars were included in the susceptibility tests. Furthermore, 10 differential genotypes and near-isogenic lines (NILs) carrying known *Lr* genes and APR effects were used as positive controls during seedling-stage rust resistance tests (Table 1). These controls facilitated the reliable characterization of the resistance responses across the tested materials.

**Table 1:** Pathotypes, near-isogenic lines (NIL)/differentials, and susceptible checks used for leaf rust against wheat materials.

*Pt* pathotype	Near-isogenic lines (NIL)/differential	Susceptible check
11(0R8), 12–2(1R5), 12–3(49R37), 12–5(29R45), 12–7(93R45), 77–1(109R63), 77–5(121R63-1), 77–7(121R127), 77–8(258R31), 77–9(121R60-1), 77–10(377R60-1), 104–1(21R31-1), 104–2(21R55) and 104–4(93R57)	Malakoff *Lr*1, Democrat *Lr*3, Tc* *Lr*10, Tc* *Lr*13, *Lr*19, IWP 94 *Lr*23+, Tc* *Lr*23, *Lr*24, Benno *Lr*26 and *Lr*34	Local Red, Agra Local

### Rust pathotypes

Fourteen distinct *Pt* pathotypes, collected from various wheat-growing regions of India, were utilized in greenhouse experiments to investigate seedling resistance and characterize *Lr* genes. These pathotypes represent all major groups of leaf rust pathogens, including the most virulent and prevalent races from groups 77 and 104 (Table 1). The avirulence/virulence patterns of these *Pt* races on the known *Lr* genes are shown in Supplementary Table S2. The pure inoculum of these *Pt* pathotypes was initially provided by the ICAR-Indian Institute of Wheat and Barley Research (IIWBR), Regional Station, Flowerdale, Shimla, and Himachal Pradesh. To ensure the availability of fresh urediospores for experimental use, the obtained inoculum was multiplied individually with the highly susceptible wheat cultivar A-9-30-1 under controlled greenhouse conditions. The multiplication process followed the standard protocols described in previous studies (Singh et al., 2020).

### Seedling stage resistance tests

#### Plant materials and growth conditions

Seedling stage resistance tests were conducted on 86 experimental wheat materials, including two susceptible checks and 10 near-isogenic lines (NILs)/differentials carrying known *Lr* genes. The resistance of these materials against 14 distinct Pt pathotypes was evaluated. The experiments were performed under controlled conditions in temperature-regulated growth chambers or greenhouses at two locations: the ICAR-Indian Institute of Wheat and Barley Research (IIWBR), Regional Station, Flowerdale, Shimla; and the National Phytotron Facility, ICAR-Indian Agricultural Research Institute (IARI), New Delhi.

#### Seedling establishment and inoculation

Each test material was represented by 4–5 seeds sown in aluminum trays measuring 11 × 4 × 3 inches. The trays were filled with sterilized fine-loamy soil autoclaved at 60°C for 1 h to eliminate microbial contaminants. The soil was enriched with farmyard manure at a 3:1 ratio and supplemented with 5 g NPK fertilizer (12:32:16 ratio) to ensure optimal seedling growth. The seedlings were cultivated in a spore-free greenhouse environment maintained at a temperature of 25–30°C, relative humidity of 50–70%, and a 12-h photoperiod to simulate ideal growing conditions.

At the two-leaf stage, seven-day-old seedlings were inoculated using a spray gun. Urediospores of each *Pt* pathotype were suspended in light mineral oil (Soltrol 170^®^, Chevron Phillips Chemicals Asia Pvt. Ltd., Singapore) at a concentration of 2–3 mg/mL, corresponding to 5–10 × 10^3^ spores/mL. This suspension ensured the uniform coverage and effective delivery of the inoculum. Following inoculation, the seedlings were incubated for 48 h in a humid chamber maintained at 20°C, 100% relative humidity, and a 12-h photoperiod to promote fungal infection and establishment. After the incubation period, the inoculated plants were transferred to a greenhouse environment maintained at a temperature of 20 ± 2°C, relative humidity of 80–90%, a 12-h photoperiod, and illumination intensity of approximately 15,000 lux (Bhardwaj et al., 2010). These controlled conditions ensured consistent disease development and facilitated accurate assessment of resistance responses in the test materials.

#### Disease scoring

Fourteen days post-inoculation, the infection types (ITs) were assessed for each seedling using a modified Stakman scale (Stakman et al., 1962), as described by Bhardwaj et al. (2010). The ITs were categorized into distinct classes to differentiate resistance levels: ITs of 0 to “–” indicated no visible symptoms or hypersensitive flecks, reflecting a highly resistant response; ITs of 1 to 2 represented minute uredia with necrosis, indicating resistance; an IT of 2+ denoted small to medium uredia with chlorotic halos, indicating moderate resistance; IT 3 corresponded to large uredia with or without chlorosis and profuse sporulation, suggesting moderate susceptibility; and an IT of 3 + indicated the presence of both 3+ and 3+ pustules, characteristic of susceptibility to highly susceptible responses. Seedling tests were conducted three times under uniform conditions to ensure accuracy and resolve any inconsistencies, enabling the robust and reliable characterization of resistance phenotypes across the tested materials.

### Characterization of *Lr* genes

The identification and characterization of *Lr* genes in the test materials were achieved through an integrated approach combining gene-matching techniques, IT patterns, and data from multiple patho-phenotyping studies. Further refinement of resistance gene profiles was accomplished by analyzing genetic interactions among various R genes, specific IT responses, genetic backgrounds of the genotypes, and associated morphological markers. Leaf tip necrosis (LTN) was used as a key phenotypic indicator, complemented by IT matrices, gene-matching methods, and multi-pathotype data to identify *Lr*1, *Lr*10, *Lr*13, *Lr*23, *Lr*26, and *Lr*34. The presence of *Lr*1 was confirmed based on its incompatible reaction with 12 distinct *Pt* pathotypes, demonstrating its effectiveness against these races. The *Lr*10 gene exhibited resistance to pathotypes 12–5, 16–1, and 77 but was susceptible to other tested pathotypes. Similarly, *Lr*13 conferred resistance to pathotypes 12–2, 16–1, and 77 while showing susceptibility to the remaining pathotypes. The *Lr*23 gene displayed resistance to infection when exposed to pathotypes 16–1 and 77 but was ineffective against other tested races. *Lr*10 demonstrated vulnerability to most pathotypes, except for 12–5, 16–1, and 77, against which it provided resistance. Likewise, *Lr*13 was effective against races 12–2, 16–1, and 77, whereas *Lr*23 exhibited a resistant reaction specifically against races 16–1 and 77, remaining susceptible to other races. In the case of *Lr*26, certain races are virulent, but this gene elicits immunity against races 12–2, 16–1, 77, and 77–2 (Bhardwaj et al., 2010).

### Field-based evaluation for adult plant resistance

#### Experimental locations

Field experiments were conducted at the Wheat Rust Pathology Experiment Farm, ICAR-Indian Agricultural Research Institute (IARI), New Delhi, during two consecutive rabi seasons (2020–21 and 2021–22). The objective was to assess and phenotype the same set of 86 test materials, along with two susceptibility checks, for adult plant resistance (APR) against leaf rust under artificially induced epiphytic conditions. The experimental farm is geographically located at 28°40′23″N latitude and 77°13′27″E longitude, at an elevation of 228.61 meters above sea level. This site falls within a semi-arid, subtropical climate zone characterized by clay loam and alluvial soil textures, making it ideal for wheat cultivation and disease screening. Additionally, this location serves as a well-established wheat rust resistance screening center in the Union Territory of the Delhi region, which is part of the North-Western Plain Zone (NWPZ) of India. Detailed meteorological data recorded on a standard weekly basis during the study period are presented in Supplementary Tables S3, S4. These data confirmed that environmental conditions were accurately correlated with disease development and resistance evaluations.

#### Experimental design and plot establishment

The field experiment was conducted using a randomized block design (RBD) with three replicates to ensure statistical robustness and minimize experimental error. Each wheat genotype was planted in small adjacent plots consisting of two rows, each 1 m in length, with a row spacing of 25 cm. The distance between individual plots was maintained at 50 cm to prevent cross-contamination and ensure uniformity in the disease assessment. Planting was carried out during the first week of December for both rabi seasons (2020–21 and 2021–22), adhering to the optimal sowing practices for wheat cultivation in the region. Standard agricultural practices, including timely irrigation, fertilization, and weed management, were strictly followed to maintain consistent crop growth and development. Irrigation channels were established between replicates to ensure uniform water distribution across the experimental site. Additionally, to create intense and continuous disease pressure under field conditions, the experimental plots were bordered with two rows of highly susceptible wheat cultivars, namely Local Red and A-9-30-1. These border rows served as a source of inoculum, facilitating the uniform spread of *Pt* pathotypes and ensuring the reliable evaluation of APR in the test materials.

#### Inoculation with *Pt* pathotypes

To artificially induce epiphytotic conditions in the field, a balanced mixture of the four most prevalent *Pt* pathotypes (12–5, 77–5, 77–9, and 104–2) representing all major virulence groups was utilized. The urediospore suspension was prepared by suspending spore dust in water supplemented with a few drops of Tween-20 to ensure uniform dispersal and adherence to the plant surfaces. Inoculation of the infector rows, consisting of highly susceptible wheat cultivars, was performed during the last week of January each year. The suspension was applied evenly onto susceptible infector rows using a hand sprayer in the evening to maximize the infection efficiency under favorable environmental conditions. Following inoculation, the foliar canopy was kept moist by applying fine droplets of water until nightfall, ensuring prolonged leaf wetness to facilitate fungal penetration and establishment. This procedure ensured consistent and intense disease pressure across the experimental plots, enabling reliable evaluation of APR in the test materials.

#### Disease assessment

Slow-rusting APR was evaluated by analyzing the host response and quantifying various epidemiological parameters, including the coefficient of infection (CI), final disease severity (FDS), relative area under the disease progress curve (rAUDPC), and apparent infection rate (*r*). Disease severity and ITs were assessed six times at seven-day intervals between February 24th and March 30th during each study year (2020–21 and 2021–22). Disease scoring commenced when susceptibility checks exhibited 25–30% severity across all replicates for each genotype/plot. Disease severity was scored using the modified Cobb scale (Peterson et al., 1948), whereas adult plant infection types were categorized as follows: R (resistant), MR (moderately resistant), MS (moderately susceptible), or S (susceptible) (Roelfs et al., 1992). The CI value was calculated by multiplying the disease severity by a constant factor assigned to each infection type: immune = 0, R = 0.2, MR = 0.4, M = 0.6, MS = 0.8, and S = 1 (Stubs et al., 1986). AUDPC and rAUDPC were estimated from multiple disease severity measurements using the established methodologies described by Milus (1986). Briefly, the AUDPC was calculated as the sum of the trapezoidal areas under the disease progress curve, whereas the rAUDPC was derived by normalizing the AUDPC of each genotype against that of the susceptible check and expressed as a percentage. The apparent infection rate (*r*) was determined using the method outlined by Vanderplank (1963), which quantifies the rate of disease development over time based on disease scores recorded at successive time points.

#### Categorization of APR levels

The 86 test materials were categorized into three distinct levels of adult plant resistance (APR): high, moderate, and low, based on their disease response and the values of key epidemiological parameters, including CI, FDS, rAUDPC, and *r*. These parameters were evaluated under field conditions to assess the durability and effectiveness of slow-rusting resistance for each genotype. Categorization was performed using standardized criteria outlined in Supplementary Table S5 (Singh et al., 2020), which provided clear thresholds for classifying genotypes into the respective APR categories.

#### Molecular analysis of *Lr* genes

Genomic DNA was isolated from fresh leaves of individual plants at the 3–4 leaf stage using the cetyltrimethylammonium bromide (CTAB) extraction method for all 86 wheat materials (Doyle and Doyle, 1990). The concentration and purity of the extracted DNA were assessed by 0.8% agarose gel electrophoresis, with lambda uncut DNA serving as a standard. The quality and quantity of the DNA samples were confirmed using a NanoDrop™ Lite Spectrophotometer (Thermo Fisher Scientific Inc., Waltham, MA, United States). The DNA samples were subsequently diluted to a working concentration of 25 ng/μL and stored at −20°C for further analysis. To detect and confirm the presence or absence of specific *Lr* genes, three gene-based molecular markers closely linked to known *Lr* genes (*Lr10, Lr24, and Lr34*) were used (Table 2). Polymerase chain reaction (PCR) was performed for each target gene using the corresponding molecular markers under optimized conditions. The amplified PCR products were separated on 4% MetaPhor agarose gels at a constant voltage of 80 volts for 2 h to ensure clear resolution of the bands. This molecular analysis facilitated the precise identification of *Lr* genes in the test materials, providing critical insights into their genetic resistance profiles.

**Table 2:** List of primers used for detection of leaf rust resistance (*Lr*) genes.

*Lr* gene	Marker type	Primer	Sequence of primer (5′ → 3′)	Annealing temp. (°C)	Size (bp)	Chr.	Reference
*Lr*10	SSR (Dominant)	F1.2245 Lr10-6/r2	F: 5’ GTGTAATGCATGCAGGTTCC 3′ R: 5′ AGGTGTGAGTGAGTTATGTT 3’	58	289	1AS	Schachermayr et al. (1997)
*Lr*24	SSR (Codominant)	Xbarc71	F: 5’GCGCTTGTTCCTCACCTGCTCATA3′ R: 5’GCGTATATTCTCTCGTCTTCTTGTTGGTT3’	57	+100 −120	6BL	Schachermayr et al. (1995)
*Lr*34	SSR (Codominant)	csLV34	F: 5’GTTGGTTAAGACTGGTGATGG3’ R: 5’TGCTTGCT ATTGCTGAATAGT3’	55	+150 −229	7DS	Lagudah et al. (2006)

### Statistical analysis

The recorded data were subjected to statistical analysis using SPSS software (version 16.0) to evaluate significant differences among the tested wheat genotypes for parameters associated with slow-rusting APR. To further explore the relationships between variables, Pearson correlation coefficient matrices were computed to assess the strength and significance of correlations among epidemiological parameters, including the CI, FDS, rAUDPC, and r. This comprehensive statistical approach ensured a robust interpretation of the data and facilitated the identification of genotypes exhibiting durable resistance under field conditions.

## Results

### Seedling resistance and *Lr* genes characterization

**Figure 2:**
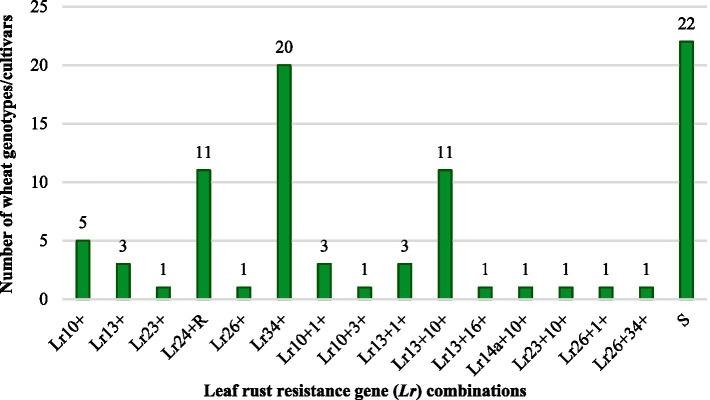
Seedling resistance genes characterized in Indian wheat genotypes/cultivars to leaf rust.

The IT patterns recorded during seedling resistance tests, along with the postulated *Lr* genes for the 86 test wheat materials, susceptible checks, and reference near-isogenic lines carrying known *Lr* genes, are summarized in Supplementary Table S6. By comparing the differential ITs observed in the test cultivars with those of isogenic lines harboring known *Lr* genes, nine potentially valuable *Lr* genes were identified (*Lr*1, *Lr*3, *Lr*10, *Lr*14a, *Lr*16, *Lr*23, *Lr*24, *Lr*26, and *Lr*34). These genes were characterized either individually or in combination with other resistance genes in the 86 genotypes (Figure 2 and Table 3). Among the identified genes, *Lr*10 was the most frequently detected, and was present in 22 genotypes. The non-race-specific APR gene *Lr*34 was found in 21 genotypes and was confirmed through molecular marker analysis and the presence of leaf tip necrosis (LTN), a morphological indicator, in combination with IT matrices. The gene *Lr*13 was postulated in 18 genotypes, whereas *Lr*24 was identified in 11 genotypes. The remaining genes, *Lr*1, *Lr*3, *Lr*14a, *Lr*16, *Lr*23, and *Lr*26, were detected in eight, one, one, one, two, and three genotypes, respectively. In seedling-stage rust resistance tests, some cultivars exhibited resistance to all 14 *Pt* pathotypes, whereas others showed susceptibility similar to susceptible checks. Overall, the majority of the test cultivars were susceptible to one or more of the prevalent Pt pathotypes. However, their potential to confer slow-rusting APR was further evaluated under field conditions to determine their effectiveness in mitigating leaf rust at later growth stages. This comprehensive characterization of *Lr* genes at the seedling stage provides critical insights into the genetic basis of resistance in the tested materials, laying the foundation for subsequent evaluations of durable resistance under natural epiphytotic conditions.

**Table 3:** Inferred presence of the *Lr* gene(s) in Indian wheat collection.

S. no.	Genotype/Cultivar	*Lr* gene(s)	S. no.	Genotype/Cultivar	*Lr* gene(s)
1	NP 4	S	44	PBW 54	*Lr*13 + 10+
2	NP 100	S	45	RAJ 1482	*Lr*13 + 10+
3	NP 111	*Lr*24 + R	46	SAGARIKA	*Lr*14a + 10+
4	NP 12	*Lr*24 + R	47	UP 2121	*Lr*10+
5	NP 52	S	48	DL153-2 (KUNDAN)	S
6	NP 165	*Lr*10+	49	GW 120	S
7	C 591	S	50	HD 2307	S
8	NP 710	*Lr*10+	51	HUW 213	*Lr*34+
9	NP 718	S	52	J 405	*Lr*34+
10	NP 745	S	53	TAWA 267	*Lr*13+
11	NP 760	*Lr*24 + R	54	WH 291	*Lr*23 + 10+
12	NP 761	*Lr*13+	55	K 7410	*Lr*13+
13	KENPHAD 25	*Lr*24 + R	56	BW 11	*Lr*34+
14	HY 12	S	57	K 8020	*Lr*13 + 10+
15	NP 770	*Lr*34+	58	PBW 120	*Lr*34+
16	HY 5	*Lr*34+	59	PBW 138	*Lr*34+
17	HYB 11	S	60	UP 1109	*Lr*13 + 1+
18	C 281	*Lr*34+	61	HI 977	*Lr*34+
19	C 286	*Lr*34+	62	HS 240	*Lr*10 + 1+
20	C 285	*Lr*24 + R	63	HP 1633	*Lr*34+
21	LERMA RAJO	*Lr*13 + 1+	64	HS 295	S
22	CHHOTI LERMA	*Lr*13 + 16+	65	PBN 51	*Lr*13 + 10+
23	PV 18	*Lr*10+	66	DL 784–3 (VAISHALI)	*Lr*13 + 10+
24	SHARBATI SONORA	*Lr*23+	67	PBW 299	*Lr*13 + 10+
25	LALBAHADUR	*Lr*13 + 10+	68	HP 1731	*Lr*34+
26	GW 10	*Lr*26 + 34+	69	K 8962 (INDRA)	*Lr*34+
27	D 134	*Lr*13 + 10+	70	DL 788–2 (VIDISHA)	*Lr*13 + 10+
28	K 816	S	71	DDK 1009 (GANGA)	*Lr*34+
29	J 1–7	*Lr*13 + 10+	72	HS 365	*Lr*26 + 1+
30	WL 711	*Lr*34+	73	HW 1085	*Lr*24 + R
31	HS 1138-6-4 (SHAILJA)	*Lr*10 + 1+	74	NW 1014	S
32	UP 262	*Lr*34+	75	SONAK	*Lr*13 + 1+
33	WL 410	*Lr*10 + 3+	76	HI 1454	*Lr*24 + R
34	HP 1102	*Lr*13 + 10+	77	KRL 19	*Lr*34+
35	HUW 12 (MALVIA 12)	*Lr*13 + 10+	78	PBW 396	*Lr*34+
36	IWP 72	*Lr*14a + 10+	79	K 9162	*Lr*24 + R
37	KSML 3	*Lr*10+	80	HUW 510	*Lr*13 + 1+
38	UP 115	S	81	HW 2045	*Lr*24 + R
39	AJANTA	S	82	K 7903	*Lr*10 + 1+
40	HW 517	S	83	MP 4010	*Lr*10+
41	MLKS 11	*Lr*34+	84	RAJ 4037	S
42	UP 2003	*Lr*34+	85	WR 544	*Lr*26+
43	WL 1562	*Lr*13+	86	HI 1500	S

### Evaluation for APR under field conditions

The slow-rusting APR of 86 test materials and two susceptible checks was evaluated based on host plant reactions and epidemiological parameters (CI, FDS, rAUDPC, and r) under field conditions (Supplementary Table S7). Analysis of variance revealed highly significant variation (*p* < 0.01) among genotypes, including susceptible checks, for all APR-related parameters during both cropping seasons, 2020–21 and 2021–22 (Supplementary Table S8). More than 64 genotypes exhibited quantitative, non-race-specific APR, despite susceptibility to prevalent *Pt* pathotypes at the seedling stage. These findings underscore the importance of APR as a durable resistance mechanism. The positive correlations among FDS, CI, rAUDPC, and r confirmed their utility in assessing slow-rusting APR.

#### Final disease severity

**Figure 3:**
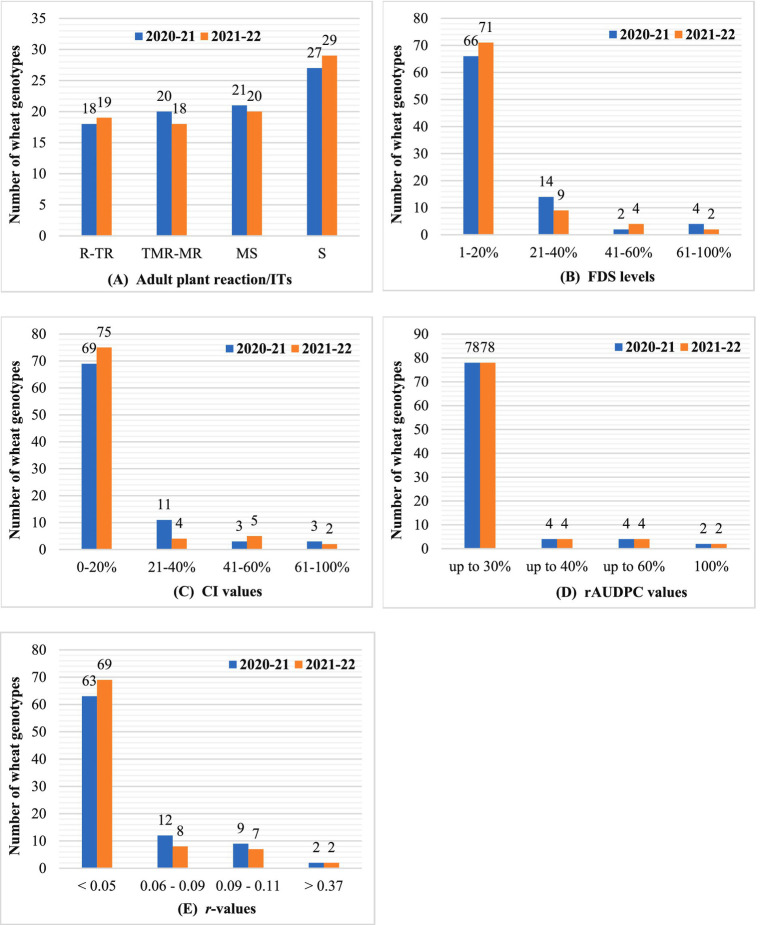
Graphical representation of the overall performance of the 86 wheat materials assessed for adult plant slow rusting resistance against leaf rust based on the values of different APR parameters recorded in the field conditions: **(A)** host response/adult plant reaction/ITs; **(B)** final disease severity (FDS); **(C)** coefficient of infection (CI); **(D)** rAUDPC; **(E)** apparent infection rate (*r*).

A marked increase in disease pressure was observed during both rabi seasons, with susceptible checks exhibiting the highest severity levels. In 2020–21, susceptible checks reached 95–100% FDS, reflecting extreme susceptibility. Adult plant infection responses revealed diverse resistance profiles: 18 genotypes showed ‘R-TR’ (resistant to moderately resistant), 20 had ‘TMR-MR’ (moderately resistant), and 21 displayed ‘MS’ (moderately susceptible) reactions (Figure 3A and Supplementary Table S3). Genotypes with ‘MS’ or ‘MR’ responses are likely to carry genes for all-stage or slow-rusting adult plant resistance (APR) (Singh et al., 2005). Based on the FDS thresholds, genotypes were categorized into high (1–20% FDS), moderate (21–40%), and low (41–60%) APR levels (Singh et al., 2020). Notably, 66 genotypes exhibited high APR, 14 showed moderate APR, and two had low APR (Figure 3B and Supplementary Table S7). In 2021–22, disease patterns mirrored the previous season, although the severity slightly declined (Figure 3B and Supplementary Table S7). Susceptible checks reached 90–95% FDS. Three genotypes (NP 111, NP 12 and HI 1500) improved from ‘TR’ to ‘R’ ITs, while two (GW 10 and MP 4010) shifted from ‘MS’ to ‘S’ (susceptible), highlighting the variability in resistance stability.

#### Coefficient of infection

The CI integrates both host response and FDS to quantify resistance levels in adult plants. Genotypes were classified into four categories based on CI values: high APR (CI ≤ 20), moderate APR (CI ≤ 40), low APR (CI ≤ 60), and high susceptibility (CI > 60), following established thresholds (Ali et al., 2009; Singh et al., 2020). In the 2020–21 growing season, susceptible checks exhibited extreme disease severity (95–100% FDS), resulting in correspondingly high CI values (Figure 3C and Supplementary Table S7). Of the 86 genotypes evaluated, 69 showed high adult plant resistance (CI ≤ 20), 11 exhibited moderate APR (CI ≤ 40), three displayed low APR (CI ≤ 60), and three were classified as highly susceptible (CI > 60). In the 2021–22 season, the CI trends remained consistent with those observed in the previous year, although disease pressure was slightly reduced (Figure 3C and Supplementary Table S7). Susceptible checks reached 90–95% FDS, yet the overall categorization of genotypes remained stable across both seasons. This consistency underscores the reliability of CI as a robust metric for evaluating APR under variable environmental conditions and reinforces its utility in resistance breeding programs.

#### Relative area under the disease progress curve

The rAUDPC quantifies disease progression over time, with lower values indicating slower epidemic development. Genotypes were categorized into high (≤30% of susceptibility check), moderate (≤40%), and low (≤60%) APR levels (Schachermayr et al., 1997; Tong et al., 2024). In 2020–21, 78 genotypes, including 18 with ‘R-TR’ infection types (ITs), exhibited rAUDPC values ≤30%, reflecting high slow-rusting APR (Figure 3D and Supplementary Table S7). These genotypes likely harbor APR genes, as their low rAUDPC values contrast with late-season ITs, suggesting delayed disease progression (Parlevliet, 1988). In 2021–22, the results remained consistent, with the rAUDPC values aligned with the previous season (Figure 3D and Supplementary Table S7). This stability highlights rAUDPC as a robust metric for evaluating durable resistance, particularly for identifying genotypes with slow-epidemic traits that are critical for long-term disease management.

#### Apparent infection rate

It measures disease progression over time, with lower values indicating a slower epidemic spread. In 2020–21, all genotypes exhibited *r*-values lower than those of susceptible checks (0.37–0.38). Notably, 63 genotypes showed minimal disease progression (*r* < 0.05), reflecting high APR (Figure 3E and Supplementary Table S7). Susceptible (S) genotypes had the highest *r*-values (0.21–0.23), whereas moderately susceptible (MS) genotypes displayed the lowest (0.02–0.08). High APR genotypes were defined as those with *r* < 0.05, moderate APR as 0.06–0.09, aligning with other resistance metrics (Ali et al., 2007; Singh et al., 2020). In 2021–22, the trends mirrored the prior season, with consistent *r*-value categorizations (Figure 3E and Supplementary Table S7). This consistency reinforces the reliability of *r*-values for identifying genotypes with durable resistance, particularly those exhibiting slow epidemic progression that is critical for APR breeding strategies.

#### Categorization for slow-rusting APR levels

**Figure 4:**
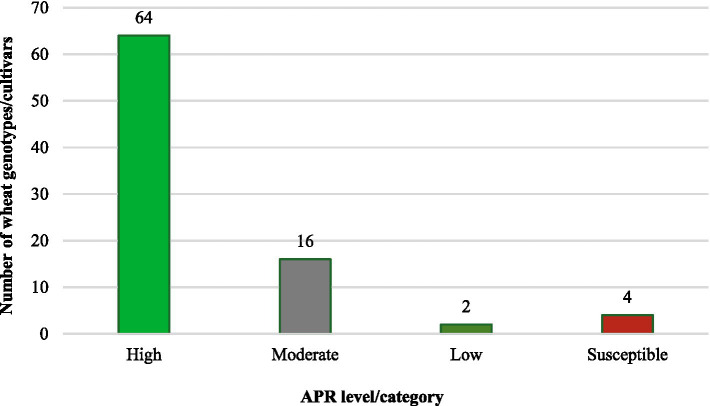
Promising levels of slow-rusting adult plant resistance identified against leaf rust in wheat genotypes/cultivars.

The 86 test genotypes and two susceptible controls were categorized into three APR levels (high, moderate, and low) based on the adult plant infection type and integrated analysis of slow-rusting components (CI, rAUDPC, FDS, and *r*-values). Across both the 2020–21 and 2021–22 seasons, 64 genotypes consistently exhibited high APR, 16 showed moderate APR, and 2 displayed low APR (Figure 4 and Table 4).

**Table 4:** Promising slow-rusting adult plant resistance characterized against leaf rust in wheat genotypes (*rabi* seasons 2020–21 and 2021–22).

APR level/category	Number	Indian wheat genotype/cultivar
High	64	NP 4, NP 100, NP 111, NP 12, NP 52, NP 165, NP 710, NP 718, NP 745, NP 760, NP 761, KENPHAD 25, HYB 11, C 281, C 286, C 285, LERMA RAJO, CHHOTI LERMA, PV 18, SHARBATI SONORA, LALBAHADUR, D 134, J 1–7, WL 711, HS 1138-6-4 (SHAILJA), UP 262, HP 1102, HUW12 (MALVIA 12), IWP 72, KSML 3, MLKS 11, WL 1562, PBW 54, RAJ 1482, SAGARIKA, UP 2121, DL153-2 (KUNDAN), J 405, TAWA 267, BW 11, K 8020, PBW 120, PBW 138, UP 1109, HI 977, HS 240, HP 1633, PBN 51, DL 784–3 (VAISHALI), PBW 299, HP 1731, K8962 (INDRA), DL788-2 (VIDISHA), DDK 1009 (GANGA), HS 365, HW 1085, SONAK, HI 1454, KRL 19, HW 2045, K 7903, MP 4010, WR 544 and HI 1500
Moderate	16	C 591, NP 770, HY 5, GW 10, WL 410, UP 115, AJANTA, HW 517, UP 2003, GW 120, HD 2307, HUW 213, WH 291, K 7410, K 9162 and HUW 510
Low	02	HY 12 and HS 295
Susceptible	04	K 816, NW 1014, PBW 396 and RAJ 4037

Notably, all genotypes classified as high/moderate APR were susceptible to one or more *Pt* pathotypes at the seedling stage, confirming the presence of non-race-specific, slow-rusting APR genes expressed exclusively at the adult plant stage. This distinction underscores the utility of these genotypes for durable resistance breeding, as APR genes often confer broad-spectrum long-lasting protection against dynamic pathogen population.

#### Association among slow-rusting APR components

**Figure 5:**
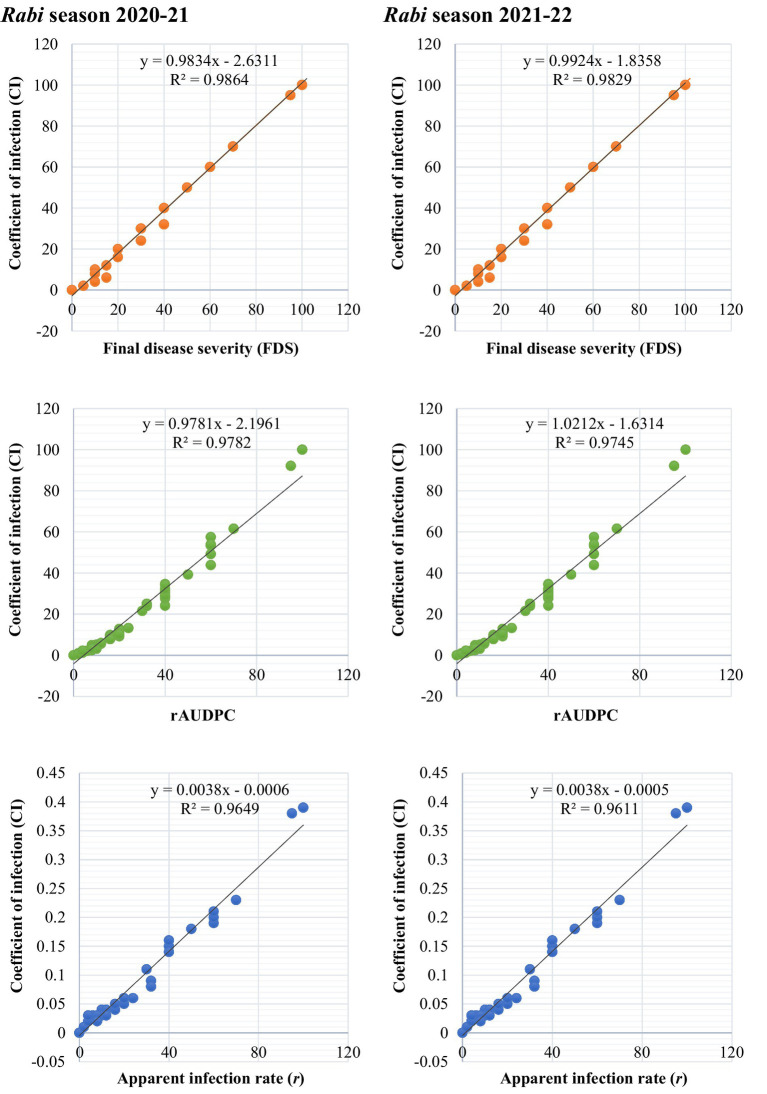
Association between slow-rusting adult plant resistance parameters to leaf rust across 86 wheat genotypes/cultivars and two susceptible checks during *rabi* seasons 2020–22.

**Figure 6:**
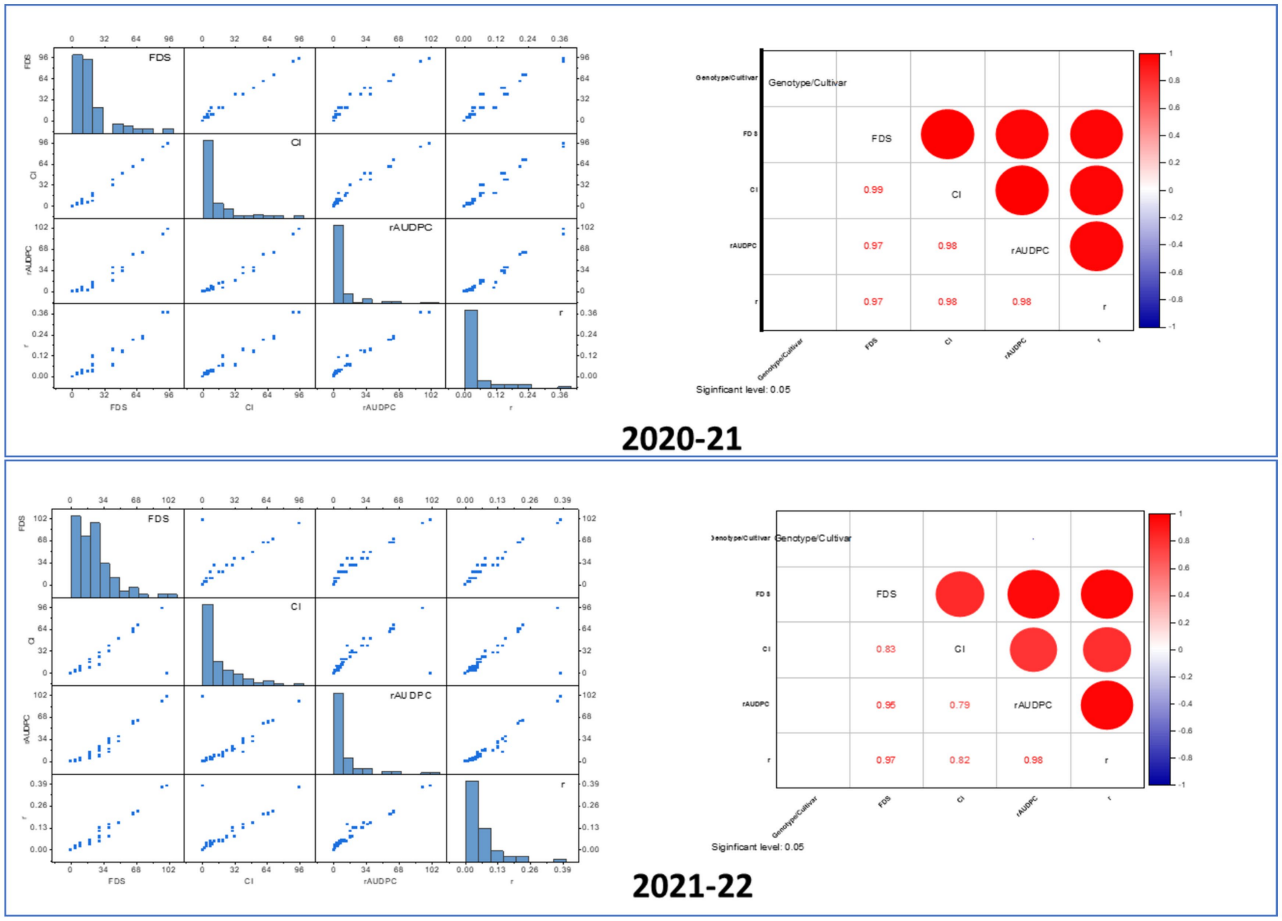
Correlation and distribution of wheat leaf rust resistance metrics (FDS, CI, rAUDPC) in adult plants. In the scatter plot matrix, the distribution of each variable shows how different *Lr* genes impact the adult plant response to infection. Highly resistant plants (due to effective *Lr* genes) would have low values in metrics like FDS, CI, and rAUDPC, which would show a skewed distribution in the histograms (toward lower values). Strong correlations between different resistance metrics (CI, rAUDPC, FDS) would indicate that plants with strong APR tend to perform consistently across these measurements. Red and blue color in the correlation matrix indicates positive and negative correlation among resistance matrices, respectively. The strong positive correlations in the heatmap imply that higher FDS correlates with higher values in related disease severity metrics, such as CI and rAUDPC. The correlation matrix shows that CI is strongly correlated with other disease metrics, indicating that plants with high infection severity will likely have high CI as well. A low rAUDPC value indicates slower disease development and is typically associated with the presence of effective *Lr* genes in wheat. In the scatter plot, rAUDPC would correlate strongly with FDS and CI, as shown by the positive correlations in the heatmap. Wheat lines with effective resistance genes would exhibit low values across these parameters (low rAUDPC, low CI, low FDS), forming a tightly clustered group of points in the scatter plot.

**Figure 7:**
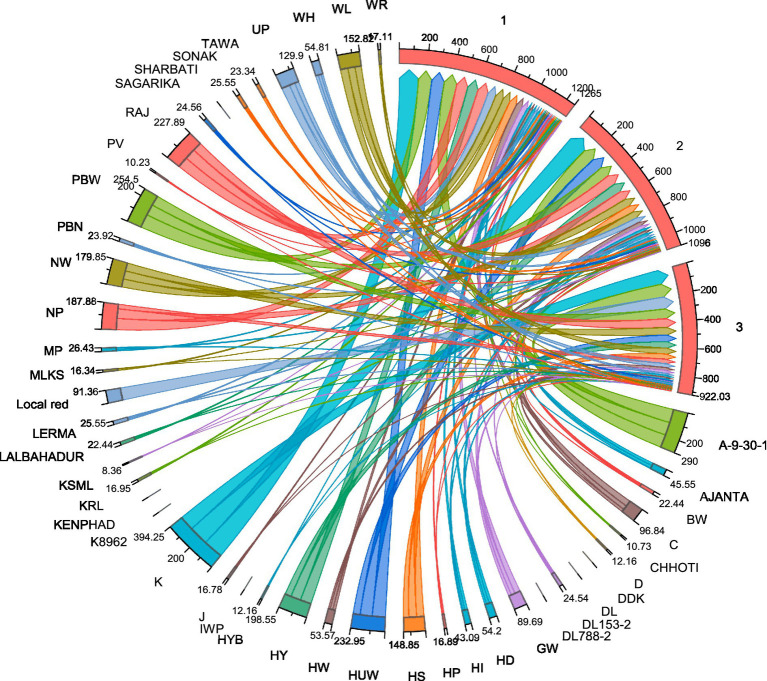
Chord diagram illustrating the relationships between wheat genotypes and leaf rust resistance responses. The chord diagram represents the interaction between different wheat genotypes (labeled around the circumference) and their associated leaf rust resistance metrics. Each colored chord reflects the relationship between wheat cultivars and the severity of leaf rust based on parameters such as final disease severity (FDS), coefficient of infection (CI), and relative area under the disease progress curve (rAUDPC). Thicker and more intense lines correspond to higher disease susceptibility, while thinner or fewer lines indicate stronger resistance in certain genotypes. The diagram also highlights the clustering of genotypes that share similar resistance profiles, potentially linked to the presence of specific *Lr* genes. This visual representation aids in understanding the distribution of resistance traits among the wheat cultivars, providing insights into the genetic diversity and potential for breeding programs aimed at improving leaf rust resistance.

To evaluate the relationships between slow-rusting adult plant resistance (APR) metrics, correlations among key epidemiological parameters—coefficient of infection (CI), final disease severity (FDS), relative area under the disease progress curve (rAUDPC), and disease progress rate (r)—were analyzed, with CI considered the primary parameter because of its comprehensive representation of disease intensity (Singh et al., 2020; Ali et al., 2009). In the 2020–21 growing season, CI exhibited strong positive correlations with FDS (*R*^2^ = 0.9838), rAUDPC (*R*^2^ = 0.9781), and *r*-values (*R*^2^ = 0.9649), indicating that these components collectively reflect the disease progression dynamics in wheat genotypes (Figure 5). Similar correlations were observed during the 2021–22 season, reinforcing the stability and consistency of these relationships across the different growing environments (Figure 5). The persistent correlation patterns highlight the reliability of the CI as a composite metric for assessing slow-rusting APR. The integration of multiple disease parameters into a unified evaluation framework is visually depicted through a scatter plot matrix (Figure 6) and chord diagram (Figure 7), which facilitates the interpretation of complex relationships among metrics and enables streamlined resistance evaluation in breeding programs. By consolidating multiple disease parameters into a single reliable metric, such as CI, breeders can more effectively identify genotypes with durable resistance, contributing to the development of climate-resilient wheat varieties. This approach not only enhances the precision of resistance screening but also supports the efficient deployment of APR genes in future breeding efforts.

### Molecular characterization of *Lr* genes

In this study, three molecular markers tightly linked to the leaf rust resistance genes *Lr*10, *Lr*24, and *Lr*34 were employed to screen and validate the presence of these genes in a panel of 86 wheat genotypes (Supplementary Figures X1–X3 and Supplementary Table S9). These markers facilitated precise molecular detection, enabling confirmation of resistance gene profiles across the tested materials. This approach underscores the utility of marker-assisted selection in characterizing genetic resistance to leaf rust, which is a critical step toward enhancing disease resilience in wheat breeding programs.

#### PCR-based detection of *Lr*10

The *Lr*10-specific molecular marker is a dominant marker, producing a 289 bp amplicon, confirming the presence of the resistance gene. Among the 86 wheat genotypes screened, 24 (27.9%) tested positive for *Lr*10 (Supplementary Table S9 and Supplementary Figure X2). This subset of genotypes harboring *Lr*10 highlights its prevalence and potential utility in resistance breeding.

#### PCR-based detection of *Lr*24

The molecular marker Xbarc71, tightly linked to the *Lr24* resistance gene, generated a 100 bp amplicon, indicating the presence of the functional allele, while the 120 bp fragment indicates the recessive allele. This marker behaves as a codominant marker, positioned 2.4 cM from the *Lr*24 locus, making it a reliable tool for marker-assisted selection. Screening of the 85 wheat genotypes revealed that 16 (18.8%) carried *Lr*24 (Supplementary Table S9 and Supplementary Figure X2). These findings validate Xbarc71 as an effective marker for identifying *Lr*24-carrying genotypes, which are critical for enhancing leaf rust resistance in wheat breeding initiatives.

#### PCR-based detection of *Lr*34

*Lr*34 is a non-race-specific APR gene that plays a critical role in imparting durable resistance to leaf rust in wheat. Amplification of *Lr*34 yielded two distinct band sizes: 150 bp (presence of the resistant allele, *Lr*34) and 229 bp (presence of the recessive allele). In certain cases, both bands appeared, indicating heterozygosity (Lagudah et al., 2006). The positive control ‘Baxter’ consistently amplified the 150 bp band, confirming the presence of *Lr*34. Of the 86 genotypes tested, 33 (38.4%) were found to carry the 150 bp band, thereby confirming the presence of the *Lr*34 gene (Supplementary Table S9 and Supplementary Figure X3).

#### Genotypes carrying multiple *Lr* genes

Several of the tested genotypes carried combinations of two or more *Lr* genes. Three genotypes (UP 1109, HS 1138-6-4, and K 8962) were identified as carriers of all three resistance genes (*Lr*10, *Lr*24, and *Lr*34). These genotypes exhibited consistently low final disease severity (FDS ≤ 15%), coefficient of infection (CI ≤ 15), and relative area under the disease progress curve (rAUDPC ≤ 25%) across both years of field evaluation, indicating strong slow-rusting resistance. Other dual-gene combinations, *Lr*24 *+ Lr*34 (C 286 and WR 544), *Lr*10 *+ Lr*34 (D 134, HP 1102, HUW 12, IWP 72, UP 2121, DL 153–2, PBW 299, HP 1731, HS 365, and HW 1085) and *Lr*10 *+ Lr*24 (K 8020 and C 285) also showed improved resistance compared to single-gene carriers. These genotypes demonstrated reduced disease progression and lower AUDPC values than those carrying only one of the genes. The apparent infection rate (*r*) was also significantly reduced in lines with multiple *Lr* genes, indicating slower epidemic development and improved resistance stability over time. These results highlight the importance of gene pyramiding in enhancing the durability and effectiveness of resistance, particularly under high disease pressure. The integration of the major seedling resistance genes (*Lr*10 and *Lr*24) with durable APR genes (*Lr*34) provides a robust defense mechanism against evolving pathogen races.

## Discussion

Rust diseases caused by *Puccinia* spp. are among the most economically damaging biotic constraints on global wheat production (Malysheva et al., 2023). While host resistance continues to be the most sustainable and cost-effective management strategy, the rapid evolution of pathogen virulence poses significant challenges to the durability of race-specific resistance genes such as *Lr*1, *Lr*3, *Lr*10, *Lr*23, *Lr*24, and *Lr*26. This study highlights the importance of integrating molecular marker-assisted selection with phenotypic screening and epidemiological analysis to identify and deploy durable resistance mechanisms in Indian wheat germplasm.

Although major effects of seedling resistance genes offer strong protection under controlled conditions, their limited longevity under field conditions necessitates a shift toward combinations of qualitative and quantitative resistance (Singh et al., 2015; Srinivas et al., 2023). Our findings confirmed that genotypes carrying non-race-specific APR genes, particularly *Lr*34, located on chromosome arm 7DS and linked to a multi-resistance locus (Yr18/Sr57/Pm38/Sb1/Bdv1/Ltn1) demonstrated more stable and broad-spectrum protection against evolving *Pt* pathotypes (PIB, 2024). The widespread presence of *Lr*34 in 38.37% of the tested materials suggests its critical role in long-term resistance breeding programs, especially when pyramided with complementary genes, such as *Lr*10 and *Lr*24 (Savary et al., 2019). This gene confers broad-spectrum resistance to yellow rust, stem rust, powdery mildew, spot blotch, and barley yellow dwarf virus with additive effects when pyramided with other resistance genes such as *Lr*12 and/or *Lr*13 (Ul Islam et al., 2023).

Molecular marker analysis confirmed the presence of *Lr*10, *Lr*24, and *Lr*34 in 24, 16, and 33 genotypes, respectively. Notably, three genotypes (UP 1109, HS 1138-6-4, and K 8962) carried all three genes and showed consistently low disease severity (FDS ≤ 15%), coefficient of infection (CI ≤ 15), and relative area under the disease progression curve (rAUDPC ≤25%) across both years of field evaluation. These triple-gene carriers exemplify the benefits of gene pyramiding, demonstrating significantly enhanced resistance compared with lines harboring only one or two of these genes. Dual-gene combinations, such as *Lr*10 *+ Lr*34 and *Lr*24 *+ Lr*34, also exhibit improved performance compared to single-gene carriers, underscoring the additive effects of combining race-specific and APR genes (Ul Islam et al., 2023). Importantly, our data showed that pyramided lines exhibited slower disease progression rates (*r*), indicating delayed epidemic development and prolonged protection during the critical grain-filling stage. This supports the hypothesis that stacking multiple resistance loci enhances the genetic complexity of defense responses, reduces selection pressure on individual pathogen races, and prolongs resistance durability. These insights align with those of earlier studies showing that gene combinations can delay resistance breakdown and improve field-level protection under high disease pressure (Singh et al., 2004; Mapuranga et al., 2022).

Despite the utility of molecular markers in gene validation, we observed discrepancies between phenotypic and genotypic data in some cases, particularly regarding the diagnostic band sizes for *Lr*24. We have clarified that Xbarc71 behaves as a codominant marker, where the presence of the 100 bp band indicates the functional allele, while the absence of the 120 bp fragment further confirms its diagnostic value (Schachermayr et al., 1995; Tong et al., 2024). Such clarifications are essential for the accurate interpretation of marker-trait associations and reproducibility in future studies. The prevalence of *Lr34* in Indian germplasms contrasts sharply with its lower frequency in global studies, such as 6.1% in Chinese cultivars and 16.7% in Central Asian germplasms (Winzeler et al., 2000). Given its enduring effectiveness against evolving Pt races, expanding the deployment of *Lr34* is vital for broadening the genetic basis for future wheat varieties (Meyer et al., 2021). Marker-assisted selection of *Lr*34, *Lr*10, and *Lr*24 can streamline breeding efforts by combining APR with race-specific resistance to achieve durable rust management (Chen et al., 2014). Partially resistant wheat genotypes, governed by multiple minor genes, offer durable protection against diverse rust races and can significantly delay epidemic onset (Nayar et al., 2003). However, their long-term efficacy may be challenged by pathogen evolution driven by factors such as long-distance migration, mutations, and selection pressure from widely deployed cultivars (Ordoñez et al., 2010).

Our study revealed strong positive correlations between the coefficient of infection (CI) and field disease severity (FDS), the relative area under the disease progression curve (rAUDPC), and the disease progression rate (*r*). These findings align with previous research on cereal rust pathosystems, highlighting the utility of low FDS values in phenotyping partial/slow-rusting APR under field conditions (Bhardwaj et al., 2021; Singh et al., 2017; Srinivas et al., 2024). Such field-based assessments are critical for developing wheat varieties with broad-spectrum, long-lasting resistance (McIntosh, 1975). By leveraging exotic and Indian germplasms as reservoirs of novel resistance alleles, these findings provide a roadmap for reducing fungicide reliance and safeguarding global food security (Herrera-Foessel et al., 2012). The integration of phenotypic and molecular data highlights the strategic value of gene pyramiding in enhancing durability and underscores the importance of deploying diverse resistance mechanisms to combat the dynamic threat of rust pathogens (Mapuranga et al., 2022). While this study focused on well-characterized *Lr* genes commonly used in marker-assisted selection, recent genomic studies (Tong et al., 2024) have highlighted the increasing number of cloned rust resistance genes in wheat. Incorporating markers for newly identified resistance loci in future screenings may further enhance the precision and durability of resistance profiling in Indian germplasm.

## Conclusion

The Indian wheat varieties in the study show a wide range of resistance to leaf rust, from strong resistance to susceptibility. Some cultivars, like HP 1761, HS 365, HUW 468, PBW 498, and VL 832, possess diverse resistance genes, with both race-specific (seedling-stage) and non-race-specific (adult plant) resistance. This highlights the importance of leveraging phenotypic and molecular data to identify diverse resistance mechanisms. The emphasis on deploying a mix of non-race-specific APR and race-specific resistance underscores the need for a holistic approach to effectively combat the dynamic threat of rust pathogens. These findings contribute significantly to the understanding of resistance dynamics in wheat and offer actionable strategies for breeding climate-resilient varieties with sustained resistance to leaf rust.

## Data Availability

The original contributions presented in the study are included in the article/Supplementary material, further inquiries can be directed to the corresponding author.
