# Supplementary material for: Multiple patho-phenotyping and molecular analysis to characterize wide-spectrum durable leaf rust resistance in wheat collections from India
Source: Front Microbiol. 2025 Jul 3;16:1596282. doi: 10.3389/fmicb.2025.1596282 (PMC12267166; doi:10.3389/fmicb.2025.1596282)
Supplement: Supplementary file 1 [file Data_Sheet_1.docx]

**Supplementary Tables (****ST)**

**Table S1.** The pedigree and origin of 86 Indian wheat materials used in the present study.

| **S.No.** | **Cultivar/genotype** | **Pedigree** | **Origin** | **Year of release** |
| --- | --- | --- | --- | --- |
| 1 | NP 4 | SEL. LOCAL MUNDA | PUSA | 1905 |
| 2 | NP 100 | MNWH/NP22 | PUSA | 1910 |
| 3 | NP 111 | MUT. NP4 | PUSA | 1910 |
| 4 | NP 12 | SEL. LOCAL MUNDA | PUSA | 1910 |
| 5 | NP 52 | NP6/6D | PUSA | 1920 |
| 6 | NP 165 | NP4/FR | PUSA | 1930 |
| 7 | C 591 | TYPE9/8B | LYALLPUR | 1934 |
| 8 | NP 710 | NP52/NP165 | NEW DELHI | 1948 |
| 9 | NP 718 | NP710'S' | NEW DELHI | 1948 |
| 10 | NP 745 | NP710'S' | NEW DELHI | 1949 |
| 11 | NP 760 | NP710'S' | NEW DELHI | 1949 |
| 12 | NP 761 | NP710'S' | NEW DELHI | 1949 |
| 13 | KENPHAD 25 | K58F(L.1/N14) | NIPHAD | 1951 |
| 14 | HY 12 | HY11 'S' | POWARKHEDA | 1952 |
| 15 | NP 770 | KNS/NP4 | NEWDELHI | 1952 |
| 16 | HY 5 | A090/WIS245 'S' | POWARKHEDA | 1953 |
| 17 | HYB 11 | A115/WIS 245'S' | POWARKHEDA | 1953 |
| 18 | C 281 | C591/NP4 | JULANDHAR | 1955 |
| 19 | C 286 | TYPE1 (DR)/KHP(DIC.)//C591/3/C250 | JULANDHAR | 1958 |
| 20 | C 285 | C228/KB256G | JULANDHAR | 1960 |
| 21 | LERMA RAJO | Y50/N10B//L52/3/2*LR | NEW DELHI | 1965 |
| 22 | CHHOTI LERMA | Lr64'S'//HUAR | NEW DELHI | 1969 |
| 23 | PV 18 | KS | LUDHIANA | 1969 |
| 24 | SHARBATI SONORA | AMBER MUT. OF SONORA 64 | NEW DELHI | 1969 |
| 25 | LALBAHADUR | CIMMYT | DURGAPURA | 1971 |
| 26 | GW 10 | S308/WS217 | JUNAGARH | 1973 |
| 27 | D 134 | RS31/WIS245'S' | DURGAPURA | 1974 |
| 28 | K 816 | CIANO'S'*(S.64*KI REND) | KANPUR | 1974 |
| 29 | J 1-7 | SELECTION FROM J1 | JUNAGARH | 1975 |
| 30 | WL 711 | NP/TOB'S'3/8156//KAL/BB | LUDHIANA | 1977 |
| 31 | HS 1138-6-4 (SHAILJA) | E48770/SONALIKA | SHIMLA | 1978 |
| 32 | UP 262 | S308/BJ66 | PANTNAGAR | 1978 |
| 33 | WL 410 | SON63/S326//KS | LUDHIANA | 1978 |
| 34 | HP 1102 | 8156(B)/NAD63 | PUSA | 1980 |
| 35 | HUW 12 (MALVIA 12) | NP876/CIANO66 | VARANASI | 1980 |
| 36 | IWP 72 | E6056/2*KS | DWR | 1980 |
| 37 | KSML 3 | ML253RON-CHA*KAL-NOR67ML265 (CNO-SON-KL.REND)*KAL2ML277 GTO-KAL*BB CNO | LUDHIANA | 1980 |
| 38 | UP 115 | NP887/E4870//UP302 | PANTNAGAR | 1980 |
| 39 | AJANTA | PW5/Y53 | BADNAPUR, MH | 1981 |
| 40 | HW 517 | BB-CC/CIANO'S'*//NO66-PJ62 | WELLINGTON | 1982 |
| 41 | MLKS 11 | CIMMYT | DWR | 1982 |
| 42 | UP 2003 | BB/2*7C | PANTNAGAR | 1982 |
| 43 | WL 1562 | KAL/JN//UP301 | LUDHIANA | 1982 |
| 44 | PBW 54 | HD2160/WG377 | LUDHIANA | 1983 |
| 45 | RAJ 1482 | NAPO/TOB'S'/8156/KAL/BB | DURGAPURA | 1983 |
| 46 | SAGARIKA | NP798/KAL | CHIPLIMA | 1983 |
| 47 | UP 2121 | UP366/SAMAKA68 | PANTNAGAR | 1984 |
| 48 | DL 153-2 (KUNDAN) | TANORI71/NP890 | NEW DELHI | 1985 |
| 49 | GW 120 | INIA66/CNO//INIA166/BB/3/Y50E/3*KAL | JUNAGARH | 1985 |
| 50 | HD 2307 | HD2160/116-1-3 | NEW DELHI | 1985 |
| 51 | HUW 213 | NORTENO/MOTI//HD2160 | VARANASI | 1985 |
| 52 | J 405 | CNO/INIA//BB/3/CNO/4/S'/PJ62//GALLO | VIJAPUR | 1985 |
| 53 | TAWA 267 | BB/7C | POWARKHEDA | 1985 |
| 54 | WH 291 | HD1925/HD832//23584 | HISAR | 1985 |
| 55 | K 7410 | K812/KAL | KANPUR | 1986 |
| 56 | BW 11 | KVZ/TI71//TZTO | MALDA | 1987 |
| 57 | K 8020 | KAL/HD1982 | KANPUR | 1987 |
| 58 | PBW 120 | WG377/HD2160 | LUDHIANA | 1987 |
| 59 | PBW 138 | RAVI43/HD2177 | LUDHIANA | 1987 |
| 60 | UP 1109 | UP262/UP368 | PANTNAGAR | 1987 |
| 61 | HI 977 | GLL/AUST61.157//CNO#66/3/Y50E/KAL | INDORE | 1988 |
| 62 | HS 240 | AU/KAL/BB/3/WOP/PAVON | SHIMLA | 1989 |
| 63 | HP 1633 | RL6010/5*SKA | PUSA | 1992 |
| 64 | HS 295 | CQT/AZ//IA555/ALDML'S'/PEL1276.79 | SHIMLA | 1992 |
| 65 | PBN 51 | BUC'S'/FLK'S' | PARBHANI | 1992 |
| 66 | DL 784-3 (VAISHALI) | KAL*4/TR38027*4/3AG3/HD2281 | NEWDELHI | 1993 |
| 67 | PBW 299 | BB/KAL//WL711/PBW65 | LUDHIANA | 1993 |
| 68 | HP 1731 | LIRA//PARULA/TONICHI | PUSA | 1995 |
| 69 | K 8962 (INDRA) | K7401/HD2160 | KANPUR | 1996 |
| 70 | DL 788-2 (VIDISHA) | K7537/HD2160MUT//HD2278/DL896-2(LR24/SR24) | NEWDELHI | 1997 |
| 71 | DDK 1009 (GANGA) | NP200*4//NP200/ALTAR84 | DHARWAD | 1998 |
| 72 | HS 365 | HS207/SONALIKA | SHIMLA | 1998 |
| 73 | HW 1085 | UNNATHKALYANSONA*2//CPAN3057 | WELLINGTON | 1998 |
| 74 | NW 1014 | HAHN'S' | FAIZABAD | 1998 |
| 75 | SONAK | LR24/SONALIKA | HISAR | 1998 |
| 76 | HI 1454 | HI1076/CC505/HI1136 | INDORE | 2000 |
| 77 | KRL 19 | PBW255/KRL1-4 | CSSRI | 2000 |
| 78 | PBW 396 | CNO67/MFD//MON'S'/3/SERI | LUDHIANA | 2000 |
| 79 | K 9162 | K7827/HD2204 | KANPUR | 2001 |
| 80 | HUW 510 | HD2278/HUW234//DL230-16 | VARANASI | 2002 |
| 81 | HW 2045 | HD2402*6/SUNSTAR*6/C-80-1 | WELLINGTON | 2002 |
| 82 | K 7903 | HD1982/K816 | KANPUR | 2002 |
| 83 | MP 4010 | ANGOSTURA88 | GWALIOR | 2003 |
| 84 | RAJ 4037 | DL788-2/RAJ3717 | DURGAPURA | 2003 |
| 85 | WR 544 | KS/HD1999//HD2204/3/DW38 | NEW DELHI | 2004 |
| 86 | HI 1500 | HW2002*2//STREMPALLI/PNC 5 | INDORE | 2006 |

**Table S2.** The binomial designation and avirulence/virulence patterns of leaf rust (*Puccinia triticina*) pathotypes on the *Lr* genes used in the study.

| **S.No.** | ***Pt* pathotype designation** | | **Avirulence/virulence pattern** |
| --- | --- | --- | --- |
|  | **Old** | **New** |  |
| 1 | 11 | 0R8 | *Lr*1, 2a, 2b, 2c, 3, 9, 10, 12, 13, 14a, 14b, 14ab, 15, 16, 17a, 17b, 18, 19, 21, 22a, 22b, 23, 24, 25, 26, 28, 29, 30, 32, 33, 34, 36,37, 38, 39, 40, 42, 43, 44, 45, 47, 48, 49/*Lr*11, 20, 27+31, 35 |
| 2 | 12-2 | 1R5 | *Lr*1, 2a, 9, 10, 13, 15, 17a, 17b, 18, 19, 20, 24, 25, 26, 28, 29, 32, 36, 39, 40, 42, 43, 45, 47/*Lr*2b, 2c, 3, 11, 12, 14a, 14b, 14ab, 16, 21, 22a, 22b, 23, 27+31, 30, 33, 34, 35, 37, 38, 44, 48, 49 |
| 3 | 12-3 | 49R37 | *Lr*1, 2a, 9, 19, 20, 23, 24, 25, 28, 29, 32, 36, 39, 42, 43, 45, 47/*Lr*2b, 2c, 3, 10, 11, 12, 13, 14a, 14b, 14ab, 15, 16, 17a, 17b, 18, 21, 22a, 22b, 26, 27, 30, 33, 34, 35, 37, 38, 40, 44, 48,49 |
| 4 | 12-5 | 29R45 | *Lr*1, 2a, 9, 10, 13, 15, 19, 24, 25, 28, 29, 32, 36, 39, 42, 43, 45, 47/*Lr*2b, 2c, 3, 11, 12, 14a, 14b, 14ab, 16, 17a, 17b, 18, 20, 21, 22a, 22b, 23, 26, 27+31, 30, 33, 34, 35, 37, 38, 40, 44, 46, 48,49 |
| 5 | 12-7 | 93R45 | *Lr*1, 2a, 9, 13, 15, 19, 24, 25, 28, 29, 32, 36, 39, 42, 43, 45, 47/*Lr*2b, 2c, 3, 10, 11, 12, 14a, 14b, 14ab, 16, 17a, 17b, 18, 20, 21, 22a, 22b, 23, 26, 27+31, 30, 33, 34, 35, 37, 38, 40, 44, 46, 48, 49 |
| 6 | 77-1 | 109R63 | *Lr*9, 17, 17a, 17b, 19, 23, 24, 25, 27+31, 28, 29, 32, 36, 39, 42, 43, 45, 47/*Lr*1, 2a, 2b, 2c, 3, 10, 11, 12, 13, 14a, 14b, 14ab, 15, 16, 18, 20, 21, 22a, 22b, 26, 30, 33, 35, 37, 38, 44, 48, 49 |
| 7 | 77-5 | 121R63-1 | *Lr*9, 19, 24, 25, 28, 29, 32, 39, 42, 43, 45, 47/*Lr*1,2a, 2b, 2c, 3,10, 11, 12,13, 14a, 14b, 14ab, 15, 16, 17a, 17b, 18, 20, 21, 22a, 22b, 23, 26, 27, 30, 33, 34, 35, 36, 37, 38, 40, 44,48, 49 |
| 8 | 77-7 | 121R127 | *Lr*18, 19, 24, 25, 28, 29, 32, 39, 40, 42, 45, 47/*Lr*1, 2a, 2b, 2c, 3, 9, 10, 11, 12, 13, 14a, 14b, 14ab, 15, 16, 17a, 17b, 20, 21, 22a, 22b, 23, 26, 27+31, 30, 33, 34, 35, 36, 37, 38, 43, 44, 48, 49 |
| 9 | 77-8 | 258R31 | *Lr*9, 23, 24, 25, 26, 27+31, 28, 29, 32, 36, 39, 45, 47/*Lr*1 2a, 2b, 2c, 3a, 10, 11, 13, 14a, 14b, 14ab, 15, 16, 17, 18, 19, 20, 21, 22a, 22b, 30, 33, 35, 37, 38, 44, 48, 49 |
| 10 | 77-9 | 121R60-1 | *Lr*2a, 2b, 2c, 9, 19, 24, 25, 28, 32, 39, 42, 45, 47/*Lr*1, 3, 10, 11, 12, 13, 14a, 14b, 14ab, 15, 16, 17a, 17b, 18, 20, 21, 22a, 22b, 23, 26, 27+31, 30, 33, 34, 35, 36, 37, 38, 44, 46, 48, 49 |
| 11 | 77-10 | 377R60-1 | *Lr*2a, 2b, 2c,9, 19, 24, 25, 32, 39, 42, 45, 47/*Lr*1, 3, 10, 11, 12, 13, 14a, 14b, 14ab, 15, 16, 17a, 17b, 18, 20, 21, 22a, 22b, 23, 26, 28, 27+31, 30, 33, 34, 35, 36, 37, 38, 44, 46, 48, 49 |
| 12 | 104-1 | 21R31-1 | *Lr*2a, 9, 15, 19, 24, 25, 26, 28, 29, 32, 39, 42, 43, 45, 47/*Lr*1, 2b, 2c, 3, 10, 11, 12, 13, 14a, 14b, 14ab, 16, 17a, 17b, 18, 20, 21, 22a, 22b, 23, 27, 30, 33, 34, 35, 36, 37, 38, 40, 44, 48, 49 |
| 13 | 104-2 | 21R55 | *Lr*9, 10, 13, 15, 19, 20, 24, 25, 28, 29, 32, 36, 39, 42, 43, 45, 47 /*Lr*1,2a, 2b, 2c, 3, 11,12, 14a, 14b, 14ab, 16, 17a, 17b, 18, 21, 22a, 22b, 23, 26, 27+31, 30, 33, 34, 35, 37, 38, 40, 44, 48,49 |
| 14 | 104-4 | 93R57 | *Lr*2a, 3, 9, 15, 19, 24, 25, 28, 32, 39, 42, 43, 45, 47/*Lr*1, 2b, 2c, 10, 11, 12, 13, 14a, 14b, 14ab, 16, 17a, 17b, 18, 20, 21, 22a, 22b, 23, 26, 27+31, 29, 30, 33, 34, 35, 36, 37, 38, 40, 44, 46, 48, 49, 51, 57 |

**Table S3.** The standard meteorological weeks average weather data at experimental farm, ICAR-IARI, New Delhi during *rabi* season from October 2020 to April 2021.

| **Year/Date** | **Week No. (SMW)** | **TEMP. (ºC)** | | | **RH (%)** | | | **RF**  **(mm)** | **BSS**  **(hrs)** | **EVP**  **(mm)** | **WS**  **(kmph)** |
| --- | --- | --- | --- | --- | --- | --- | --- | --- | --- | --- | --- |
|  |  | **Max** | **Min** | **Mean** | **Max** | **Min** | **Mean** |  |  |  |  |
| Oct. 1-7, 2020 | 40 | 35.4 | 19.8 | 76.1 | 45.7 | 0.0 | 8.9 | 6.0 | 4.3 | 35.4 | 19.8 |
| Oct. 8-14 | 41 | 34.9 | 19.8 | 81.9 | 41.6 | 0.0 | 7.3 | 5.3 | 2.6 | 34.9 | 19.8 |
| Oct. 15-21 | 42 | 34.1 | 16.2 | 82.3 | 32.0 | 0.0 | 6.7 | 4.3 | 2.0 | 34.1 | 16.2 |
| Oct. 22-28 | 43 | 32.8 | 12.9 | 85.7 | 31.4 | 0.0 | 5.5 | 3.5 | 1.9 | 32.8 | 12.9 |
| Oct. 29-Nov. 4 | 44 | 30.2 | 10.9 | 86.6 | 28.7 | 0.0 | 4.8 | 3.0 | 2.2 | 30.2 | 10.9 |
| Nov. 5-11 | 45 | 28.7 | 10.5 | 86.4 | 32.0 | 0.0 | 1.6 | 2.8 | 1.6 | 28.7 | 10.5 |
| Nov. 12-18 | 46 | 27.1 | 11.3 | 82.1 | 44.4 | 0.5 | 2.4 | 2.9 | 1.8 | 27.1 | 11.3 |
| Nov. 19-25 | 47 | 24.5 | 7.8 | 84.1 | 35.6 | 0.0 | 5.2 | 2.4 | 2.4 | 24.5 | 7.8 |
| Nov. 26-Dec. 2 | 48 | 25.0 | 9.2 | 82.3 | 34.3 | 0.0 | 6.3 | 2.7 | 2.7 | 25.0 | 9.2 |
| Dec. 3-9 | 49 | 26.4 | 9.8 | 89.6 | 48.1 | 0.0 | 5.2 | 2.4 | 1.8 | 26.4 | 9.8 |
| Dec. 10-16 | 50 | 22.8 | 10.2 | 92.1 | 60.0 | 0.1 | 4.2 | 1.7 | 3.4 | 22.8 | 10.2 |
| Dec. 17-23 | 51 | 19.8 | 3.3 | 82.6 | 40.3 | 0.0 | 6.4 | 1.9 | 3.1 | 19.8 | 3.3 |
| Dec 24-31 | 52 | 20.1 | 3.4 | 91.8 | 50.1 | 0.0 | 5.7 | 2.1 | 3.3 | 20.1 | 3.4 |
| Jan. 1-7, 2021 | 1 | 18.5 | 9.4 | 92.0 | 76.9 | 9.4 | 0.6 | 1.1 | 3.5 | 18.5 | 9.4 |
| Jan. 8-14 | 2 | 16.7 | 5.7 | 91.3 | 69.0 | 0.0 | 3.1 | 1.4 | 4.0 | 16.7 | 5.7 |
| Jan. 15-21 | 3 | 17.9 | 6.1 | 95.1 | 56.4 | 0.0 | 2.0 | 1.7 | 3.3 | 17.9 | 6.1 |
| Jan. 22-28 | 4 | 18.3 | 4.7 | 88.6 | 63.3 | 0.0 | 4.7 | 1.8 | 3.2 | 18.3 | 4.7 |
| Jan 29-Feb.4 | 5 | 22.6 | 5.4 | 82.1 | 35.7 | 0.1 | 6.7 | 2.4 | 2.4 | 22.6 | 5.4 |
| Feb. 5-11 | 6 | 23.1 | 7.1 | 89.6 | 45.1 | 0.9 | 6.9 | 2.8 | 2.6 | 23.1 | 7.1 |
| Feb. 12-18 | 7 | 27.1 | 8.5 | 90.7 | 36.3 | 0.0 | 5.5 | 3.1 | 1.3 | 27.1 | 8.5 |
| Feb. 19-25 | 8 | 27.0 | 8.0 | 95.0 | 52.0 | 0.0 | 5.2 | 3.6 | 1.2 | 27.0 | 8.0 |
| Feb. 26-Mar. 4 | 9 | 29.9 | 12.4 | 82.0 | 29.1 | 0.0 | 8.9 | 4.4 | 4.6 | 29.9 | 12.4 |
| Mar. 5-11 | 10 | 31.4 | 14.4 | 80.9 | 32.7 | 0.3 | 7.5 | 4.1 | 4.2 | 31.4 | 14.4 |
| Mar. 12-18 | 11 | 30.8 | 14.4 | 85.4 | 36.1 | 0.0 | 4.7 | 3.8 | 3.7 | 30.8 | 14.4 |
| Mar. 19-25 | 12 | 32.8 | 16.3 | 76.3 | 41.0 | 0.0 | 4.4 | 3.9 | 4.5 | 32.8 | 16.3 |
| Mar. 26-Apr. 1 | 13 | 35.1 | 17.2 | 76.3 | 34.0 | 0.0 | 7.7 | 4.7 | 6.4 | 35.1 | 17.2 |
| Apr. 2-8 | 14 | 36.0 | 16.9 | 70.4 | 29.1 | 0.0 | 8.7 | 5.6 | 5.2 | 36.0 | 16.9 |
| Apr. 9-15 | 15 | 38.0 | 18.4 | 65.4 | 23.9 | 0.0 | 9.5 | 6.7 | 4.3 | 38.0 | 18.4 |
| Apr. 16-22 | 16 | 36.9 | 18.4 | 72.1 | 38.3 | 0.7 | 7.9 | 6.9 | 6.0 | 36.9 | 18.4 |
| Apr. 23-Apr. 30 | 17 | 37.5 | 19.3 | 65.0 | 36.6 | 0.0 | 9.0 | 7.7 | 5.1 | 37.5 | 19.3 |

^*^SMW = Standard meteorological weeks, Temp = Temperature, Max = Maximum, Min = Minimum, RH = Relative humidity, RF = Rainfall, SS = Sunshine, EVP = Evaporation and WS = Wind speed (Source: Weather Observatory, Division of Agricultural Physics, ICAR-Indian Agricultural Research Institute, New Delhi)

**Table S4.** The standard meteorological weeks average weather data at experimental farm, ICAR-IARI, New Delhi during *rabi* season from October 2021 to April 2022.

| **Year/Date** | **Week No. (SMW)** | **TEMP. (ºC)** | | | **RH (%)** | | | **RF**  **(mm)** | **BSS**  **(hrs)** | **EVP**  **(mm)** | **WS**  **(kmph)** |
| --- | --- | --- | --- | --- | --- | --- | --- | --- | --- | --- | --- |
|  |  | **Max** | **Min** | **Mean** | **Max** | **Min** | **Mean** |  |  |  |  |
| Oct. 1-7, 2021 | 40 | 34.5 | 24.6 | 91 | 66 | 5.1 | 7.6 | 3.9 | 2.6 | 34.5 | 24.6 |
| Oct. 8-14 | 41 | 35.0 | 20.5 | 88 | 48 | 0.0 | 8.7 | 4.3 | 2.9 | 35.0 | 20.5 |
| Oct. 15-21 | 42 | 31.0 | 18.7 | 88 | 63 | 7.8 | 5.8 | 3.5 | 3.4 | 31.0 | 18.7 |
| Oct. 22-28 | 43 | 29.8 | 15.9 | 87 | 61 | 5.3 | 8.1 | 3.4 | 3.0 | 29.8 | 15.9 |
| Oct. 29-Nov. 4 | 44 | 29.4 | 13.6 | 93 | 55 | 0.0 | 7.2 | 3.4 | 2.0 | 29.4 | 13.6 |
| Nov. 5-11 | 45 | 28.1 | 12.0 | 93 | 50 | 0.0 | 2.8 | 2.4 | 1.7 | 28.1 | 12.0 |
| Nov. 12-18 | 46 | 26.3 | 9.3 | 91 | 51 | 0.0 | 3.7 | 2.3 | 1.2 | 26.3 | 9.3 |
| Nov. 19-25 | 47 | 26.5 | 9.6 | 88 | 57 | 0.0 | 5.8 | 2.6 | 1.9 | 26.5 | 9.6 |
| Nov. 26-Dec. 2 | 48 | 25.4 | 10.0 | 93 | 54 | 0.3 | 4.0 | 2.0 | 1.3 | 25.4 | 10.0 |
| Dec. 3-9 | 49 | 23.7 | 10.3 | 90 | 65 | 0.0 | 4.1 | 2.1 | 1.9 | 23.7 | 10.3 |
| Dec. 10-16 | 50 | 22.2 | 6.2 | 88 | 61 | 0.0 | 4.4 | 1.7 | 1.3 | 22.2 | 6.2 |
| Dec. 17-23 | 51 | 19.6 | 4.4 | 91 | 57 | 0.0 | 5.3 | 1.6 | 3.2 | 19.6 | 4.4 |
| Dec 24-31 | 52 | 20.6 | 6.3 | 93 | 73 | 1.0 | 2.1 | 1.5 | 1.5 | 20.6 | 6.3 |
| Jan. 1-7, 2022 | 1 | 19.9 | 7.2 | 93 | 76 | 11.6 | 3.2 | 1.6 | 2.2 | 19.9 | 7.2 |
| Jan. 8-14 | 2 | 17.1 | 8.5 | 93 | 76 | 3.9 | 1.5 | 1.6 | 3.8 | 17.1 | 8.5 |
| Jan. 15-21 | 3 | 15.7 | 7.5 | 92 | 75 | 1.0 | 1.2 | 1.5 | 2.7 | 15.7 | 7.5 |
| Jan. 22-28 | 4 | 15.5 | 7.4 | 93 | 81 | 3.8 | 1.3 | 1.3 | 3.7 | 15.5 | 7.4 |
| Jan 29-Feb.4 | 5 | 19.8 | 7.4 | 90 | 72 | 0.3 | 5.5 | 2.0 | 5.3 | 19.8 | 7.4 |
| Feb. 5-11 | 6 | 21.1 | 7.5 | 91 | 65 | 1.5 | 5.6 | 2.2 | 2.6 | 21.1 | 7.5 |
| Feb. 12-18 | 7 | 24.6 | 6.9 | 90 | 37 | 0.0 | 8.7 | 3.6 | 2.8 | 24.6 | 6.9 |
| Feb. 19-25 | 8 | 25.3 | 10.5 | 91 | 40 | 2.1 | 6.7 | 3.3 | 4.8 | 25.3 | 10.5 |
| Feb. 26-Mar. 4 | 9 | 25.5 | 11.0 | 83 | 46 | 0.3 | 8.1 | 3.3 | 4.6 | 25.5 | 11.0 |
| Mar. 5-11 | 10 | 28.1 | 13.0 | 88 | 37 | 0.0 | 7.6 | 3.4 | 4.3 | 28.1 | 13.0 |
| Mar. 12-18 | 11 | 32.4 | 16.5 | 83 | 43 | 0.0 | 8.4 | 4.8 | 3.9 | 32.4 | 16.5 |
| Mar. 19-25 | 12 | 35.8 | 18.3 | 76 | 45 | 0.0 | 7.7 | 5.5 | 3.7 | 35.8 | 18.3 |
| Mar. 26-Apr. 1 | 13 | 37.4 | 16.8 | 68 | 24 | 0.0 | 9.0 | 5.4 | 4.3 | 37.4 | 16.8 |
| Apr. 2-8 | 14 | 39.1 | 16.8 | 69 | 11 | 0.0 | 9.7 | 6.1 | 3.6 | 39.1 | 16.8 |
| Apr. 9-15 | 15 | 40.8 | 20.1 | 65 | 13 | 0.0 | 8.0 | 6.4 | 3.6 | 40.8 | 20.1 |
| Apr. 16-22 | 16 | 39.9 | 21.3 | 69 | 17 | 0.0 | 8.0 | 6.8 | 4.2 | 39.9 | 21.3 |
| Apr. 23-Apr. 30 | 17 | 40.9 | 21.2 | 57.7 | 19.1 | 0.0 | 9.0 | 7.8 | 4.9 | 40.9 | 21.2 |

^*^SMW = Standard meteorological weeks, Temp = Temperature, Max = Maximum, Min = Minimum, RH = Relative humidity, RF = Rainfall, SS = Sunshine, EVP = Evaporation and WS = Wind speed (Source: Weather Observatory, Division of Agricultural Physics, ICAR-Indian Agricultural Research Institute, New Delhi)

**Table S5.** The categorization of slow rusting/partial/adult plant resistance level based on the values of different epidemiological parameters (Singh et al., 2020).

| **Category/level of APR** | **Values of different epidemiological parameters** | | | |
| --- | --- | --- | --- | --- |
|  | **FRS level (%)** | **CI value** | **rAUDPC** | ***r* value** |
| High | 1-20% | 0-20 | up to 30% of the susceptible check | less than 0.05 |
| Moderate | 21-40% | 21-40 | up to 40% of the susceptible check | 0.06 to 0.09 |
| Low | 41-60% | 41-60 | up to 60% of the susceptible check | 0.09 to 0.11 |
| Susceptible | 61-100% | 61-100 | 100% | more than 0.37 |

**Table S6.** The seedling infection types (ITs) and presence/absence of leaf rust resistance (*Lr*) genes in Indian wheat cultivars/genotypes based on gene postulation using 14 different *P. triticina* pathotypes.

| **S.No.** | **Cultivar/Genotype** | **Seedling infection types (ITs)^1^ to *Pt* pathotypes** | | | | | | | | | | | | | | ***Lr* gene** |
| --- | --- | --- | --- | --- | --- | --- | --- | --- | --- | --- | --- | --- | --- | --- | --- | --- |
|  |  | **11** | **12-2** | **12-3** | **12-5** | **12-7** | **77-1** | **77-5** | **77-7** | **77-8** | **77-9** | **77-10** | **104-1** | **104-2** | **104-4** |  |
| 1 | NP 4 | ;1 | 3+ | 3+ | 3+ | 3+ | 3+ | 3+ | 77-7 | 77-8 | 77-9 | 77-10 | 3+ | 3+ | 3+ | S |
| 2 | NP 100 | 2 | 3+ | 3+ | 3+ | 3+ | 3+ | 3+ | 3+ | 3+ | 3+ | 3+ | 3+ | 3+ | 3+ | S |
| 3 | NP 111 | ;- | ;1 | 0; | 0; | ;- | 0; | ;1  1P2 | 3+ | 3+ | 3+ | 3+ | ;1 | 0; | ;- | *Lr*24+R |
| 4 | NP 12 | ;- | 0; | 0; | 0; | ; | ; | 1P;  1P3 | 0; | 0; | 0; | 0; | ; | ;- | 0; | *Lr*24+R |
| 5 | NP 52 | 3+ | 3+ | 3+ | 3+ | 3+ | 3+ | 3+ | 0; | ;- | 0; | ;- | 3+ | 3+ | 3+ | S |
| 6 | NP 165 | ;1 | 3+ | 3+ | 2 | 3+ | 2 | 3+ | 0; | 3+ | 3+ | 3+ | 3+ | 3+ | 3+ | *Lr*10+ |
| 7 | C 591 | 0; | 3+ | 3+ | 3+ | 3+ | 3+ | 3+ | 1P0; | 3+ | 3+ | 3+ | 3+ | 3+ | 3+ | S |
| 8 | NP 710 | 0; | 3+ | 3+ | 2 | 3+ | 1P2 | 3+ | 3 | 3 | 3+ | 3+ | 3+ | 3+ | 3+ | *Lr*10+ |
| 9 | NP 718 | ;1 | 3+ | 3+ | 3+ | 3+ | 3+ | 3+ | 2P0 | 3+ | 3+ | 3+ | 3+ | 3+ | 3+ | S |
| 10 | NP 745 | 23 | 3+ | 3+ | 3+ | ;1 | 3+ | 3+ | 0; | 3+ | 3 | 3+ | 3+ | 3+ | 3+ | S |
| 11 | NP 760 | ;- | 0; | 0; | ;- | 0; | ;- | 1P0; | 0; | 3+ | 3+ | 3+ | 0; | 0; | ;- | *Lr*24+R |
| 12 | NP 761 | 0; | 2P2  1P3+ | 0;  1P3+ | 3+ | 3+ | 3+ | 3+ | 0; | 0; | 0; | ;- | 3+ | 3+ | 3+ | *Lr*13+ |
| 13 | KENPHAD 25 | ;- | 0; | ;- | 0; | ;- | ;- | 0; | 0; | 0; | 3+ | 3+ | 0; | 0; | ;- | *Lr*24+R |
| 14 | HY 12 | ; | 3+ | 1P0;  2P3 | 3+ | 3+ | 1P;1  2P3 | 3+ | 0; | 0; | ;- | ;1 | 3+ | 3+ | 3+ | S |
| 15 | NP 770 | ;1 | 1P;1  2P3 | 1P;1  1P3 | 3+ | 3+ | 1P;1  2P3 | 3+ | 3+ | 3+ | 3+ | 3+ | 3 | 3+ | 3+ | *Lr*34+ |
| 16 | HY 5 | 3 | 3+ | 3+ | 3+ | 3P;1  1P3+ | 3+ | 3+ | ;1 | 3+ | 3+ | 3+ | 3+ | 1P;1  2P3+ | 3+ | *Lr*34+ |
| 17 | HYB 11 | 0; | 33+ | 3+ | 3+ | 3+ | 3+ | 3+ | 3 | 2P;  1P3+ | 3+ | 3+ | 3+ | 3+ | 3+ | S |
| 18 | C 281 | 1P;1  1P3+ | 3+ | 3+ | 3+ | 3+ | 1P2  2P3+ | 3+ | 3+ | 3+ | 3+ | 3P;  2P2+ | 3+ | 3+ | 3+ | *Lr*34+ |
| 19 | C 286 | 0; | 3 | 3+ | 3+ | 3+ | 3 | ;1  1P3+ | 33+ | 3+ | 3+ | 3+ | 3+ | 3 | 3+ | *Lr*34+ |
| 20 | C 285 | ;- | 0; | 0; | 0; | ;- | ;1 | 1P;1  1P2 | 3+ | 3 | 3+ | 3+ | ; | ;- | 0; | *Lr*24+R |
| 21 | LERMA RAJO | 0; | ;1 | ;1 | ; | 3P;  3P3+ | 2 | 3+ | 0; | ;- | ;- | 0; | 3+ | 3+ | 3+ | *Lr*13+1+ |
| 22 | CHHOTI LERMA | 0; | 0; | 0; | ;1 | 3+ | 33+ | 2P3+  1P0; | 3 | 3+ | 3+ | 3+ | 3+ | 3P;  1P3+ | 3+ | *Lr*13+16+ |
| 23 | PV 18 | 0; | 3+ | 0; | ; | 3+ | 3+ | 3+ | 0; | 3+ | 3+ | 23 | 3+ | 3+ | 3+ | *Lr*10+ |
| 24 | SHARBATI SONORA | 0; | 33+ | ;1 | ; | ; | 3+ | 3+ | 0; | 3+ | 3+ | 3+ | 3+ | 3+ | 3+ | *Lr*23+ |
| 25 | LALBAHADUR | 0; | 3 | ; | 33+ | 3+ | ;1 | 3+ | 3+ | ;1 | X | ;1 | 3+ | 3+ | 3+ | *Lr*13+10+ |
| 26 | GW 10 | 0; | ;1 | 0; | 3+ | 3+ | 3+ | 3+ | 0; | ; | 3+ | 2 | 3+ | 3+ | 3+ | *Lr*26+34+ |
| 27 | D 134 | 0; | 0; | 0; | ; | ; | 33+ | 3+ | 0; | ;1 | 3+ | 3+ | ;1  1P3 | 2 | 3+ | *Lr*13+10+ |
| 28 | K 816 | 0; | 3+ | 3+ | 3+ | 3+ | 3+ | 3+ | 0; | 0; | 3+ | 3+ | 3+ | 3P;  2P3+ | 3+ | S |
| 29 | J 1-7 | 0; | 2 | 0; | ; | 3+ | 3+ | 3+ | 3 | 3+ | 3+ | 3+ | 3+ | ;1 | 3+ | *Lr*13+10+ |
| 30 | WL 711 | 3+ | 3+ | 3+ | 3+ | 3+ | 3+ | 3+ | 3 | ; | 3+ | 3+ | 3+ | 3+ | 3+ | *Lr*34+ |
| 31 | HS 1138-6-4 (SHAILJA) | 0; | 0; | 0; | ; | 0; | 3+ | 3+ | 1P0;  2P3 | 3+ | 3+ | 3+ | 3+ | 33+ | 3+ | *Lr*10+1+ |
| 32 | UP 262 | 1P0;  1P3 | 2P0;  1P3+ | 3+ | 3+ | 3+ | 3+ | 3+ | 3+ | 3+ | 3+ | 3+ | 3+ | 3+ | 3+ | *Lr*34+ |
| 33 | WL 410 | 0; | - | 0; | 0; | ; | 3+ | 3+ | 3+ | 3+ | 3+ | 3+ | ; 1P3+ | 3+ | 0; | *Lr*10+3+ |
| 34 | HP 1102 | 0; | 0; | 0; | 0; | 0; | ;1 | 3+ | 3+ | ; | 2P; | 3+ | 23 | 3+ | 3+ | *Lr*13+10+ |
| 35 | HUW12 (MALVIA 12) | 0; | ;1 | ; | - | 3+ | 3+ | 3+ | 0 | 3+ | 3+ | 3+ | 23 | ;1 | 3+ | *Lr*13+10+ |
| 36 | IWP 72 | 0; | - | ; | 0; | 0; | 3+ | 3+ | 3+ | 3+ | 3+ | 3+ | 33+ | 3+ | 3+ | *Lr*14a+10+ |
| 37 | KSML 3 | 0; | 33+ | 3+ | 3 | 1P0; | 3+ | 3+ | 3+ | 3+ | 3+ | 3+ | 3+ | 3+ | 3+ | *Lr*10+ |
| 38 | UP 115 | 0; | 3+ | 3+ | 3+ | 3 | 3+ | 3+ | 3+ | 3+ | 3+ | - | 3+ | 3+ | 3+ | S |
| 39 | AJANTA | 0; | 3+ | 3+ | 3+ | 3+ | 3+ | 3+ | 3+ | 3+ | 3+ | 3+ | 3 | 3+ | 3+ | S |
| 40 | HW 517 | 3 | 3+ | 3+ | 3+ | 3+ | 3+ | 3+ | 3+ | 3 | 3+ | 3+ | 3+ | 3+ | 3+ | S |
| 41 | MLKS 11 | 0; | 3+ | 3 | 3+ | 3 | 33+ | 3+ | 3+ | 0; | 3+ | 3+ | 3+ | 3+ | 3+ | *Lr*34+ |
| 42 | UP 2003 | 0; | 3+ | 3+ | 3+ | 3 | 33+ | 3+ | 3+ | 3+ | 3+ | 3+ | 3+ | 3+ | 3+ | *Lr*34+ |
| 43 | WL 1562 | 0; | 2 | 0; | ; | ;1 | 3+ | 3+ | 3 | 3+ | 3+ | 3+ | ; | ;1 | 2 | *Lr*13+ |
| 44 | PBW 54 | - | 0; | 0; | 3+ | 3+ | 3+ | 0 | 0; | 0; | 3+ | 0; | 0; | ;1 | 3+ | *Lr*23+10+ |
| 45 | RAJ 1482 | - | 0; | ;1 | 2P3;  1P3+ | X | 33+ | 3+ | 0; | 0; | 3 | - | 33+ | 33+ | 3+ | *Lr*13+ |
| 46 | SAGARIKA | 1P0;  1P2 | 3+ | 3+ | 3+ | 3+ | 3+ | 3+ | 3+ | 0; | 3+ | 1P3+  1P;1 | 3+ | 3+ | 3+ | *Lr*34+ |
| 47 | UP 2121 | 0; | 0; | 0; | - | 0; | 0; | 3+ | 3 | 3+ | 3+ | 3+ | 3+ | ; | 3+ | *Lr*13+10+ |
| 48 | DL153-2 (KUNDAN) | 0; | 0; | 3 | 3+ | 3 | 33+ | 3+ | 3+ | 3+ | 3+ | 3+ | 3 | 3+ | 3+ | *Lr*34+ |
| 49 | GW 120 | 0; | 0; | 3 | 3 | 3 | 3+ | 3+ | 3+ | 3 | 3+ | 3+ | 3 | 3+ | 3+ | *Lr*34+ |
| 50 | HD 2307 | 0; | 0; | 0; | ; | 0; | 3+ | 3+ | 3 | 3 | 3+ | 3+ | 3+ | 33+ | 0; | *Lr*13+1+ |
| 51 | HUW 213 | 0; | 3+ | 3 | 3+ | 3+ | 3+ | 3+ | 3+ | 1P;  1P3+ | 3+ | 3+ | 3+ | 3+ | 3+ | *Lr*34+ |
| 52 | J 405 | 0; | ; | ; | ; | ;1 | 3+ | 3+ | 3+ | 3 | 3+ | 3 | ;1 | 2P2 | 3+ | *Lr*10+1+ |
| 53 | TAWA 267 | 0; | 3 | 3+ | 3+ | 3+ | 3 | 3+ | 3+ | 0; | 3+ | 3+ | 3+ | 3+ | 3+ | *Lr*34+ |
| 54 | WH 291 | 3 | 23 | 2 | 3+ | 3+ | 3 | 3+ | 3+ | 3 | 3+ | 3+ | 3+ | 3+ | 3+ | S |
| 55 | K 7410 | 0; | - | 0; | 0; | 0; | 3+ | 3+ | 3+ | 3+ | 3+ | 3+ | - | 33+ | 3+ | *Lr*13+10+ |
| 56 | BW 11 | 2 | 0; | 0; | 0; | ; | 0; | 1P;  1P3+ | 3+ | 0; | - | 0; | 0; | 2P3+  1P0: | 0: | *Lr*13+10+ |
| 57 | K 8020 | 0; | 0; | 0; | 0; | ; | 2 | ;1 | 3+ | 0; | 2P;1  1P3+ | ;1  1P3+ | 3+ | 3+ | 3+ | *Lr*13+10+ |
| 58 | PBW 120 | 1P;  1P2 | 1P;1  2P3 | 3+ | 3+ | 3+ | 3+ | 3+ | ; | 2 | 33+ | 2 | 3+ | 3 | 3+ | *Lr*34+ |
| 59 | PBW 138 | 1P0;  2P3 | 1P;1  2P3 | 3+ | 3+ | 3+ | 3+ | 3+ | 3+ | 33+ | 3+ | 3 | 3+ | 3+ | 3+ | *Lr*34+ |
| 60 | UP 1109 | 0; | 0; | 0; | 0; | ;1 | 3+ | 3+ | 3+ | 3+ | 3+ | 3 | 0; | 0; | 3+ | *Lr*13+10+ |
| 61 | HI 977 | 0; | 0; | 3 | 3 | 3 | 3+ | 3+ | 0; | 0; | 3+ | ; | 3+ | 3+ | 3+ | *Lr*34+ |
| 62 | HS 240 | 0; | ;1 | 2 | 0; | 3+ | 3+ | 3+ | 3+ | 3 | 3+ | 3+ | ; | 3 | 3+ | *Lr*26+1+ |
| 63 | HP 1633 | 0; | 0; | 0; | ; | ;1 | 0; | 0; | 3+ | 3+ | 3+ | ;- | ; | 0; | ;1 | *Lr*24+R |
| 64 | HS 295 | 0; | 3+ | 3+ | 3+ | 3+ | 3+ | 3+ | 0; | 0; | 0; | ;1 | 3+ | 3+ | 3+ | S |
| 65 | PBN 51 | 0; | 0; | 0; | 0; | ; | 3+ | 3+ | 3+ | 3+ | 3+ | 3+ | ; | 0; | 3+ | *Lr*13+1+ |
| 66 | DL 784-3 (VAISHALI) | 0; | 0; | ; | 0; | ; | ; | ; | 0; | 0; | 3+ | 2P;1  1P3 | 0; | 0; | 0; | *Lr*24+R |
| 67 | PBW 299 | 0; | 3+ | 3+ | 3+ | 3+ | 3+ | 3+ | 0; | 0; | ; | 0; | 1P0;  1P3 | 3+ | 3+ | *Lr*34+ |
| 68 | HP 1731 | 0; | 0; | 3 | 3 | 3 | 3+ | 3+ | 3+ | 3 | 3+ | 3+ | 3+ | 3+ | 3+ | *Lr*34+ |
| 69 | K8962 (INDRA) | 0; | 2P0; | 0; | 0; | 0; | 0; | 0; | 3+ | 3 | 3+ | 3+ | 0; | 0; | 0; | *Lr*24+R |
| 70 | DL788-2 (VIDISHA) | 0; | ; | ; | 0; | 01 | 0; | 3+ | 0; | ;1 | ; | 0; | 0; | 0; | 3+ | *Lr*13+1+ |
| 71 | DDK 1009 (GANGA) | 0; | ;1 | 0; | 0; | ;1 | 0; | 0; | 0; | 0; | ;- | 3+ | 0; | 0; | ;1 | *Lr*24+R |
| 72 | HS 365 | 0; | ;1 | 0; | 0; | ;1 | 3+ | 1P;  2P3 | ; | 0; | ; | 0; | 2P;  2P3 | 1P2  2P;1 | 3+ | *Lr*10+1+ |
| 73 | HW 1085 | 0; | 0; | ; | 0; | ; | 0; | 3+ | 3+ | ; | 3+ | 3+ | ; | ; | 3+ | *Lr*10+ |
| 74 | NW 1014 | 3+ | 3+ | 3 | 3+ | 3+ | 3+ | 3+ | 0; | 0; | 0; | 0; | 3+ | 3+ | 3+ | S |
| 75 | SONAK | 0; | - | 0; | 3+ | 3+ | 3+ | 3+ | 1P;  1P3+ | 33+ | ;1  1P3+ | 3P3+  1P;1 | 3+ | 3+ | 3+ | *Lr*26+ |
| 76 | HI 1454 | 3+ | 3 | 3+ | 3+ | 3+ | 3+ | 3+ | 0; | 0; | 3+ | 3+ | 3+ | 2P3+ | 3+ | S |
| 77 | KRL 19 | 2P0;  2P2 | 3+ | 3+ | 1P;1  1P3 | 3+ | 3+ | 3+ | 3+ | 3+ | 3+ | 3+ | 3+ | 3+ | 3+ | *Lr*34+ |
| 78 | PBW 396 | 3+ | 3+ | 3+ | 3+ | 3+ | 3+ | 3+ | 3+ | 3 | 3+ | 2P3 | 3+ | 3+ | 3+ | S |
| 79 | K 9162 | 3+ | 3+ | 3+ | 3+ | 3+ | 3+ | 3+ | 3+ | 3+ | 3+ | 3+ | 3+ | 3+ | 3+ | S |
| 80 | HUW 510 | 3+ | 3+ | 3+ | 3+ | 3+ | 3+ | 3+ | 3 | 3+ | 3+ | 3+ | 3+ | 3+ | 3+ | S |
| 81 | HW 2045 | 0; | 0; | ;1 | ;1 | ; | 0; | ; | 3+ | 0; | 3+ | 3 | ; | ; | ;1 | *Lr*24+R |
| 82 | K 7903 | 3+ | 3+ | 3+ | 3+ | 3+ | 3+ | 3+ | 0; | 0; | ;1 | ; | 3+ | 3+ | 3+ | S |
| 83 | MP 4010 | 2P0;  P3+ | 1P0;  2P3+ | 3+ | 3 | 3+ | 3+ | 3+ | 0; | 3+ | 3+ | 3+ | 3+ | 3+ | 3+ | S |
| 84 | RAJ 4037 | 3+ | 3+ | 3+ | 3+ | 3+ | 3+ | 3+ | 0; | 3+ | 3+ | 3+ | 3+ | 3+ | 3+ | S |
| 85 | WR 544 | 0; | 3+ | 3+ | 3+ | 3+ | 3+ | 3+ | 3+ | 3+ | 3+ | 3+ | 3+ | 3+ | 3 | *Lr*34+ |
| 86 | HI 1500 | ;- | ;1 | 0; | ;1 | 0; | 0; | 0; | 0; | 3 | 3+ | 3+ | 0; | ; | ;- | *Lr*24+R |
| *Susceptible check* | | | | | | | | | | | | | | | | |
| 87 | Local Red | 3+ | 3+ | 3+ | 3+ | 3+ | 3+ | 3+ | 0; | 0; | 0; | ;- | 3+ | 3+ | 3+ | S |
| 88 | A-9-30-1 | 3+ | 3+ | 3+ | 3+ | 3+ | 3+ | 3+ | 3+ | 3+ | 3+ | 3+ | 3+ | 3+ | 3+ | S |
| *Near-isogenic line (NIL)/differential* | | | | | | | | | | | | | | | | |
| 89 | Malakoff *Lr*1 | 0; | ;1 | 0; | ;1 | ; | 3+ | 3+ | 3+ | 3+ | 3+ | 3+ | 3+ | 3+ | 0; |  |
| 90 | Democrat *Lr*3 | 3+ | 3+ | 3+ | 3+ | 3+ | 3+ | 3+ | 3+ | 3+ | 3+ | 3+ | 3+ | 3+ | ;1 |  |
| 91 | Tc* *Lr*10 | 2+ | ; | 2+ | ; | 3+ | 3+ | 3+ | 3+ | 3+ | 3+ | 3+ | ; | 3+ | 0; |  |
| 92 | Tc* *Lr*13 | 2 | 3+ | 2 | 3+ | 3+ | 3+ | 3+ | 3+ | 3+ | 3+ | 3+ | 3+ | 3+ | 0; |  |
| 93 | *Lr*19 | 0; | 0; | 0; | 0; | 0; | 0; | 0; | 0; | 0; | 0; | 0; | 0; | 0; | 0; |  |
| 94 | IWP 94 *Lr*23+ | ; | 3+ | ; | 3+ | 3+ | ; | 3+ | 3+ | ;- | 3+ | 3+ | ;1 | 3+ | 2 |  |
| 95 | Tc* *Lr*23 | ; | 3+ | ; | 3+ | 3+ | 3+ | 3+ | 3+ | ; | 3+ | 3+ | 2 | 3+ | ; |  |
| 96 | *Lr*24 | ; | 0; | 0; | 0; | 0; | 0; | 0; | 0; | 0; | 0; | 0; | 0; | 0; | 0; |  |
| 97 | Benno *Lr*26 | 3+ | 3+ | 3+ | 3+ | 3+ | 3+ | 3+ | 33+ | 0; | 3+ | 3+ | 0; | 3+ | 0; |  |
| 98 | *Lr*34 | 3+ | 3+ | 3+ | 3+ | 3 | 3 | 3C | 3+ | 3 | 3+ | 3C | 3+ | 3+ | ; |  |

^1^Seedling infection types (ITs) based on Stakman et al. (1962), with modifications (Bhardwaj et al., 2010d) as 0;, ;, 1, 2 = Resistant (R), 2+ = Moderately resistant (MR), 3 = Moderately susceptible (MS), 33+ = Susceptible (S) and 3+ = Highly susceptible (HS).

**Table S7.** The adult plant reaction and mean values of different adult plant resistance parameters (FDS, CI, rAUDPC, *r*) in Indian wheat cultivars/genotypes to leaf rust (*rabi* seasons 2020-22).

| **S.No.** | **Genotype/**  **Cultivar** | **Inferred presence of**  ***Lr* gene(s)^a^** | **Adult plant reaction^b^** | | **Mean value of APR parameters^b^** | | | | | | | |
| --- | --- | --- | --- | --- | --- | --- | --- | --- | --- | --- | --- | --- |
|  |  |  |  |  | **FDS** | | **CI** | | **rAUDPC** | | ***r*** | |
|  |  |  | **2020-21** | **2021-22** | **2020-21** | **2021-22** | **2020-21** | **2021-22** | **2020-21** | **2021-22** | **2020-21** | **2021-22** |
| 1 | NP 4 | S | S | S | 10 | 5 | 10 | 5 | 3.96 | 2.16 | 0.03 | 0.02 |
| 2 | NP 100 | S | S | S | 20 | 10 | 20 | 10 | 13.59 | 5.55 | 0.05 | 0.04 |
| 3 | NP 111 | *Lr*24+R | TR | R | 0 | 0 | 0 | 0 | 0 | 0 | 0 | 0 |
| 4 | NP 12 | *Lr*24+R | TR | R | 0 | 0 | 0 | 0 | 0 | 0 | 0 | 0 |
| 5 | NP 52 | S | S | S | 10 | 10 | 10 | 10 | 3.4 | 3.08 | 0.04 | 0.03 |
| 6 | NP 165 | *Lr*10+ | S | S | 20 | 10 | 20 | 10 | 10.76 | 5.55 | 0.06 | 0.03 |
| 7 | C 591 | S | S | S | 30 | 20 | 30 | 20 | 17.00 | 14.20 | 0.11 | 0.12 |
| 8 | NP 710 | *Lr*10+ | S | S | 10 | 10 | 10 | 10 | 3.4 | 3.08 | 0.04 | 0.03 |
| 9 | NP 718 | S | S | S | 20 | 10 | 20 | 10 | 10.76 | 5.55 | 0.06 | 0.04 |
| 10 | NP 745 | S | MS | MS | 10 | 5 | 8 | 4 | 4.72 | 1.73 | 0.03 | 0.02 |
| 11 | NP 760 | *Lr*24+R | TR | TR | 0 | 0 | 0 | 0 | 0 | 0 | 0 | 0 |
| 12 | NP 761 | *Lr*13+ | MR | MR | 5 | 5 | 2 | 2 | 0.79 | 0.61 | 0.01 | 0.01 |
| 13 | KENPHAD 25 | *Lr*24+R | R | R | 0 | 0 | 0 | 0 | 0 | 0 | 0 | 0 |
| 14 | HY 12 | S | S | S | 50 | 40 | 50 | 40 | 30.86 | 28.9 | 0.16 | 0.15 |
| 15 | NP 770 | *Lr*34+ | MR | MR | 30 | 20 | 12 | 8 | 8.23 | 6.57 | 0.05 | 0.03 |
| 16 | HY 5 | *Lr*34+ | MS | MS | 40 | 40 | 32 | 32 | 18.83 | 17.65 | 0.08 | 0.07 |
| 17 | HYB 11 | S | S | S | 10 | 5 | 10 | 5 | 5.1 | 2.16 | 0.04 | 0.02 |
| 18 | C 281 | *Lr*34+ | MR | MR | 10 | 10 | 4 | 4 | 3.54 | 3.44 | 0.03 | 0.03 |
| 19 | C 286 | *Lr*34+ | MR | MR | 10 | 10 | 4 | 4 | 3.98 | 3.53 | 0.04 | 0.03 |
| 20 | C 285 | *Lr*24+R | MR | MR | 10 | 5 | 4 | 2 | 1.35 | 0.67 | 0.03 | 0.01 |
| 21 | LERMA RAJO | *Lr*13+1+ | S | S | 20 | 10 | 20 | 10 | 10.76 | 5.55 | 0.06 | 0.04 |
| 22 | CHHOTI LERMA | *Lr*13+16+ | MS | MS | 10 | 5 | 8 | 4 | 3.62 | 1.73 | 0.03 | 0.02 |
| 23 | PV 18 | *Lr*10+ | MS | MS | 5 | 5 | 4 | 4 | 1.58 | 1.23 | 0.02 | 0.01 |
| 24 | SHARBATI SONORA | *Lr*23+ | R | R | 0 | 0 | 0 | 0 | 0 | 0 | 0 | 0 |
| 25 | LALBAHADUR | *Lr*13+10+ | MS | MS | 5 | 10 | 4 | 8 | 1.58 | 4.44 | 0.02 | 0.03 |
| 26 | GW 10 | *Lr*26+34+ | MS | S | 30 | 20 | 24 | 20 | 17.23 | 13.57 | 0.08 | 0.06 |
| 27 | D 134 | *Lr*13+10+ | S | S | 10 | 5 | 10 | 5 | 5.1 | 2.16 | 0.04 | 0.02 |
| 28 | K 816 | S | S | S | 65 | 70 | 60 | 70 | 58.51 | 62.34 | 0.21 | 0.22 |
| 29 | J 1-7 | *Lr*13+10+ | MR | MR | 20 | 10 | 8 | 4 | 5.66 | 2.78 | 0.04 | 0.03 |
| 30 | WL 711 | *Lr*34+ | MS | MS | 20 | 10 | 16 | 8 | 9.12 | 5.82 | 0.06 | 0.04 |
| 31 | HS 1138-6-4 (SHAILJA) | *Lr*10+1+ | MS | MS | 10 | 5 | 8 | 4 | 3.62 | 1.73 | 0.02 | 0.01 |
| 32 | UP 262 | *Lr*34+ | MR | MR | 20 | 10 | 8 | 4 | 5.84 | 2.68 | 0.04 | 0.03 |
| 33 | WL 410 | *Lr*10+3+ | S | S | 40 | 40 | 40 | 40 | 35.13 | 38.27 | 0.15 | 0.16 |
| 34 | HP 1102 | *Lr*13+10+ | R | R | 0 | 0 | 0 | 0 | 0 | 0 | 0 | 0 |
| 35 | HUW 12 (MALVIA 12) | *Lr*13+10+ | MS | MS | 10 | 5 | 8 | 4 | 3.62 | 1.73 | 0.02 | 0.01 |
| 36 | IWP 72 | *Lr*14a+10+ | R | R | 0 | 0 | 0 | 0 | 0 | 0 | 0 | 0 |
| 37 | KSML 3 | *Lr*10+ | MR | MR | 10 | 5 | 4 | 2 | 1.81 | 1.36 | 0.02 | 0.02 |
| 38 | UP 115 | S | S | S | 30 | 20 | 30 | 20 | 23.23 | 13.57 | 0.13 | 0.06 |
| 39 | AJANTA | S | S | S | 40 | 20 | 40 | 20 | 14.6 | 5.55 | 0.15 | 0.11 |
| 40 | HW 517 | S | S | S | 30 | 20 | 30 | 20 | 23.23 | 13.57 | 0.13 | 0.06 |
| 41 | MLKS 11 | *Lr*34+ | MR | MR | 20 | 10 | 8 | 4 | 5.18 | 2.34 | 0.03 | 0.02 |
| 42 | UP 2003 | *Lr*34+ | MR | MR | 30 | 20 | 12 | 8 | 9.11 | 7.24 | 0.05 | 0.04 |
| 43 | WL 1562 | *Lr*13+ | MS | MS | 10 | 5 | 8 | 4 | 3.62 | 1.73 | 0.03 | 0.02 |
| 44 | PBW 54 | *Lr*23+10+ | MS | MS | 20 | 10 | 16 | 8 | 9.51 | 5.92 | 0.06 | 0.03 |
| 45 | RAJ 1482 | *Lr*13+ | S | S | 20 | 10 | 20 | 10 | 7.93 | 5.55 | 0.06 | 0.04 |
| 46 | SAGARIKA | *Lr*34+ | MS | MS | 20 | 10 | 16 | 8 | 9.45 | 6.56 | 0.06 | 0.03 |
| 47 | UP 2121 | *Lr*13+10+ | MS | MS | 20 | 10 | 16 | 8 | 9.96 | 6.41 | 0.05 | 0.03 |
| 48 | DL153-2 (KUNDAN) | *Lr*34+ | MS | MS | 20 | 10 | 16 | 8 | 9.89 | 6.54 | 0.05 | 0.04 |
| 49 | GW 120 | *Lr*34+ | MS | MS | 30 | 20 | 12 | 8 | 9.14 | 8.12 | 0.05 | 0.04 |
| 50 | HD 2307 | *Lr*13+10+ | S | S | 30 | 20 | 30 | 20 | 17.00 | 14.20 | 0.11 | 0.12 |
| 51 | HUW 213 | *Lr*34+ | MS | MS | 40 | 40 | 32 | 32 | 18.83 | 17.65 | 0.08 | 0.06 |
| 52 | J 405 | *Lr*10+1+ | MR | TMR | 5 | 0 | 2 | 0 | 0.56 | 0 | 0.01 | 0 |
| 53 | TAWA 267 | *Lr*34+ | MS | MS | 20 | 15 | 8 | 6 | 5.18 | 2.34 | 0.04 | 0.03 |
| 54 | WH 291 | S | S | S | 30 | 20 | 30 | 20 | 20.39 | 14.81 | 0.13 | 0.07 |
| 55 | K 7410 | *Lr*13+10+ | MS | MS | 30 | 20 | 24 | 16 | 16.76 | 12.34 | 0.07 | 0.06 |
| 56 | BW 11 | *Lr*13+10+ | MS | MS | 5 | 10 | 4 | 8 | 1.58 | 4.44 | 0.02 | 0.03 |
| 57 | K 8020 | *Lr*13+10+ | MR | TR | 5 | 0 | 2 | 0 | 0.79 | 0 | 0.01 | 0 |
| 58 | PBW 120 | *Lr*34+ | MR | MR | 10 | 10 | 4 | 4 | 4.12 | 3.98 | 0.04 | 0.03 |
| 59 | PBW 138 | *Lr*34+ | MR | MR | 20 | 10 | 8 | 4 | 6.25 | 2.74 | 0.04 | 0.02 |
| 60 | UP 1109 | *Lr*13+10+ | R | R | 0 | 0 | 0 | 0 | 0 | 0 | 0 | 0 |
| 61 | HI 977 | *Lr*34+ | MR | MR | 10 | 10 | 4 | 4 | 4.72 | 3.54 | 0.04 | 0.02 |
| 62 | HS 240 | *Lr*26+1+ | R | R | 0 | 0 | 0 | 0 | 0 | 0 | 0 | 0 |
| 63 | HP 1633 | *Lr*24+R | R | R | 0 | 0 | 0 | 0 | 0 | 0 | 0 | 0 |
| 64 | HS 295 | S | S | S | 50 | 50 | 50 | 50 | 38.43 | 38.12 | 0.16 | 0.15 |
| 65 | PBN 51 | *Lr*13+1+ | MR | MS | 20 | 10 | 8 | 8 | 6.51 | 5.92 | 0.05 | 0.03 |
| 66 | DL 784-3 (VAISHALI) | *Lr*24+R | R | R | 0 | 0 | 0 | 0 | 0 | 0 | 0 | 0 |
| 67 | PBW 299 | *Lr*34+ | MR | MR | 10 | 10 | 4 | 4 | 5.12 | 3.74 | 0.04 | 0.03 |
| 68 | HP 1731 | *Lr*34+ | MR | MR | 20 | 10 | 8 | 4 | 6.45 | 2.89 | 0.04 | 0.02 |
| 69 | K8962 (INDRA) | *Lr*24+R | R | R | 0 | 0 | 0 | 0 | 0 | 0 | 0 | 0 |
| 70 | DL788-2 (VIDISHA) | *Lr*13+1+ | R | R | 0 | 0 | 0 | 0 | 0 | 0 | 0 | 0 |
| 71 | DDK 1009 (GANGA) | *Lr*24+R | R | R | 0 | 0 | 0 | 0 | 0 | 0 | 0 | 0 |
| 72 | HS 365 | *Lr*10+1+ | R | R | 0 | 0 | 0 | 0 | 0 | 0 | 0 | 0 |
| 73 | HW 1085 | *Lr*10+ | R | R | 0 | 0 | 0 | 0 | 0 | 0 | 0 | 0 |
| 74 | NW 1014 | S | S | S | 65 | 60 | 65 | 60 | 62.14 | 59.85 | 0.22 | 0.21 |
| 75 | SONAK | *Lr*26+ | S | S | 20 | 10 | 20 | 10 | 7.93 | 5.55 | 0.06 | 0.04 |
| 76 | HI 1454 | S | S | S | 20 | 10 | 20 | 10 | 10.76 | 5.55 | 0.06 | 0.04 |
| 77 | KRL 19 | *Lr*34+ | MR | MR | 20 | 10 | 8 | 4 | 6.68 | 2.95 | 0.04 | 0.02 |
| 78 | PBW 396 | S | S | S | 70 | 60 | 70 | 60 | 62.52 | 58.12 | 0.23 | 0.21 |
| 79 | K 9162 | S | S | S | 40 | 50 | 40 | 50 | 30.46 | 32.84 | 0.13 | 0.15 |
| 80 | HUW 510 | S | S | S | 40 | 50 | 40 | 50 | 29.23 | 32.57 | 0.13 | 0.14 |
| 81 | HW 2045 | *Lr*24+R | R | R | 0 | 0 | 0 | 0 | 0 | 0 | 0 | 0 |
| 82 | K 7903 | S | MS | MS | 10 | 5 | 8 | 4 | 3.62 | 1.73 | 0.03 | 0.02 |
| 83 | MP 4010 | S | MS | S | 20 | 10 | 16 | 10 | 9.06 | 6.43 | 0.05 | 0.03 |
| 84 | RAJ 4037 | S | S | S | 65 | 70 | 65 | 70 | 59.51 | 62.34 | 0.21 | 0.23 |
| 85 | WR 544 | *Lr*34+ | MR | MR | 20 | 10 | 8 | 4 | 7.25 | 3.11 | 0.04 | 0.03 |
| 86 | HI 1500 | *Lr*24+R | TR | R | 0 | 0 | 0 | 0 | 0 | 0 | 0 | 0 |
| *Susceptible check* | | | | | | | | | | | | |
| 87 | Local Red |  | S | S | 95 | 90 | 95.00 | 90.00 | 92.35 | 91.36 | 0.37 | 0.37 |
| 88 | A-9-30-1 |  | S | S | 100 | 95 | 100.00 | 95.00 | 100 | 100 | 0.38 | 0.37 |
|  | SE(m)± |  |  |  | 2.89 | 2.64 | 2.38 | 2.49 | 1.95 | 1.39 | 0.012 | 0.013 |
|  | LSD (5%) |  |  |  | 8.13 | 7.39 | 7.19 | 6.32 | 5.11 | 4.89 | 0.032 | 0.039 |

^a^The presence of *Lr* gene(s) are postulated in the seedling stage resistance evaluation tests conducted in the present investigations. ^b^Adult plant infection types based on Roelfs *et al.* (1992) as TR=Trace Responses; R=Resistant, presence of hypersensitive necrotic or chlorotic flecks but no uredinia; MR=Moderately Resistant, small pustules surrounded by necrotic areas; MS=Moderately Susceptible, medium sized pustules, no visible necrosis but there is chlorosis and S=Susceptible, large pustules, no necrosis or chlorosis. ^b^Data based on weekly disease observations and means of three replications.

**Table S8.** Analysis of variance for APR parameters among 86 Indian wheat genotypes/cultivars and two susceptible checks for leaf rust (*rabi* seasons 2020-22).

| **Source of variation** | **D.f.** | **Mean square value for APR parameters*** | | | | | | | |
| --- | --- | --- | --- | --- | --- | --- | --- | --- | --- |
|  |  | **FDS** | | **CI** | | **rAUDPC** | | ***r*** | |
|  |  | **2020-21** | **2021-22** | **2020-21** | **2021-22** | **2020-21** | **2021-22** | **2020-21** | **2021-22** |
| Cultivars | 85 | 2584.26** | 2496.64** | 2686.39** | 2586.13** | 2511.34** | 2421.14** | 0.34** | 0.33** |
| Replications | 2 | 221.24 | 218.98 | 114.18 | 114.12 | 150.21 | 149.21 | 0.28 | 0.26 |
| Cultivars x Replications | 170 | 21.15 | 20.91 | 16.24 | 15.64 | 4.36 | 3.78 | 0.76 | 0.68 |
| Total error | 257 | 804.11 | 802.36 | 853.11 | 851.92 | 782.74 | 763.24 | 0.13 | 0.12 |

*FDS = Final disease severity, CI = Coefficient of infection, rAUDPC = relative Area under the disease progress curve, *r* = Apparent infection rate; D.f. = degree of freedom; **indicate significant difference at P<0.01 and P<0.05

**Table S9.** The presence and absence of the three leaf rust resistance genes, *Lr*10, *Lr*24, and *Lr*34 in 86 tested wheat materials using molecular marker analysis.

| S.No. | Genotype/Cultivar | Leaf rust resistance gene | | | Number of genes* |
| --- | --- | --- | --- | --- | --- |
|  |  | *Lr*10 | *Lr*24 | *Lr*34 |  |
| 1 | NP 4 | - | - | - | 0 |
| 2 | NP 100 | - | - | - | 0 |
| 3 | NP 111 | - | + | - | 1 |
| 4 | NP 12 | - | + | - | 1 |
| 5 | NP 52 | - | - | - | 0 |
| 6 | NP 165 | + | - | - | 1 |
| 7 | C 591 | - | - | - | 0 |
| 8 | NP 710 | + | - | - | 1 |
| 9 | NP 718 | - | - | - | 0 |
| 10 | NP 745 | - | - | - | 0 |
| 11 | NP 760 | - | + | - | 1 |
| 12 | NP 761 | - | - | - | 0 |
| 13 | KENPHAD 25 | - | + | - | 1 |
| 14 | HY 12 | - | - | - | 0 |
| 15 | NP 770 | - | - | + | 1 |
| 16 | HY 5 | - | - | + | 1 |
| 17 | HYB 11 | - | - | - | 0 |
| 18 | C 281 | - | - | + | 1 |
| 19 | C 286 | - | + | + | 2 |
| 20 | C 285 | + | + | - | 2 |
| 21 | LERMA RAJO | - | - | - | 0 |
| 22 | CHHOTI LERMA | - | - | + | 1 |
| 23 | PV 18 | + | - | - | 1 |
| 24 | SHARBATI SONORA | - | - | - | 0 |
| 25 | LALBAHADUR | - | - | + | 1 |
| 26 | GW 10 | - | - | + | 1 |
| 27 | D 134 | + | - | + | 2 |
| 28 | K 816 | - | - | - | 0 |
| 29 | J 1-7 | - | - | - | 0 |
| 30 | WL 711 | - | - | + | 1 |
| 31 | HS 1138-6-4 (SHAILJA) | + | + | + | 3 |
| 32 | UP 262 | - | - | + | 1 |
| 33 | WL 410 | + | - | - | 1 |
| 34 | HP 1102 | + | - | + | 2 |
| 35 | HUW12 (MALVIA 12) | + | - | + | 2 |
| 36 | IWP 72 | + | - | + | 2 |
| 37 | KSML 3 | - | - | - | 0 |
| 38 | UP 115 | - | - | - | 0 |
| 39 | AJANTA | - | - | - | 0 |
| 40 | HW 517 | - | - | - | 0 |
| 41 | MLKS 11 | - | - | + | 1 |
| 42 | UP 2003 | - | - | + | 1 |
| 43 | WL 1562 | - | - | - | 0 |
| 44 | PBW 54 | + | - | - | 1 |
| 45 | RAJ 1482 | - | - | - | 0 |
| 46 | SAGARIKA | - | - | + | 1 |
| 47 | UP 2121 | + | - | + | 0 |
| 48 | DL153-2 (KUNDAN) | + | - | + | 0 |
| 49 | GW 120 | - | - | + | 1 |
| 50 | HD 2307 | + | - | - | 1 |
| 51 | HUW 213 | - | - | + | 0 |
| 52 | J 405 | + | - | - | 1 |
| 53 | TAWA 267 | - | - | + | 1 |
| 54 | WH 291 | - | - | - | 0 |
| 55 | K 7410 | + | - | - | 1 |
| 56 | BW 11 | + | - | - | 1 |
| 57 | K 8020 | + | + |  | 1 |
| 58 | PBW 120 | - | - | + | 1 |
| 59 | PBW 138 | - | - | + | 1 |
| 60 | UP 1109 | + | + | + | 3 |
| 61 | HI 977 | - | - | + | 1 |
| 62 | HS 240 | - | - | - | 0 |
| 63 | HP 1633 | - | + | - | 1 |
| 64 | HS 295 | - | - | - | 0 |
| 65 | PBN 51 | - | - | - | 0 |
| 66 | DL 784-3 (VAISHALI) | - | + | - | 1 |
| 67 | PBW 299 | + | - | + | 2 |
| 68 | HP 1731 | + | - | + | 2 |
| 69 | K8962 (INDRA) | + | + | + | 3 |
| 70 | DL788-2 (VIDISHA) | - | - | - | 0 |
| 71 | DDK 1009 (GANGA) | - | + |  | 1 |
| 72 | HS 365 | + | - | + | 2 |
| 73 | HW 1085 | + | - | + | 2 |
| 74 | NW 1014 | - | - | - | 0 |
| 75 | SONAK | - | - | - | 0 |
| 76 | HI 1454 | - | - | - | 0 |
| 77 | KRL 19 | - | - | + | 1 |
| 78 | PBW 396 | - | - | - | 0 |
| 79 | K 9162 | - | - | - | 0 |
| 80 | HUW 510 | - | - | - | 0 |
| 81 | HW 2045 | - | + | - | 1 |
| 82 | K 7903 | - | - | - | 0 |
| 83 | MP 4010 | - | - | - | 0 |
| 84 | RAJ 4037 | - | - | - | 0 |
| 85 | WR 544 | - | + | + | 2 |
| 86 | HI 1500 | - | + | - | 1 |

(+) Fragment is amplified and the gene is present; (−) no specific fragment is amplified and the gene is absent.

*Number of genes detected in that particular genotype.


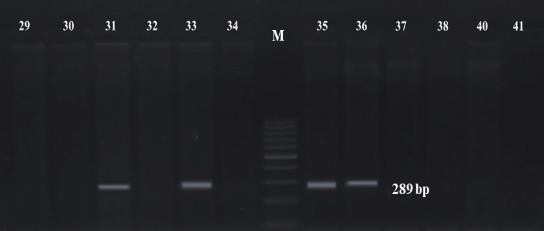

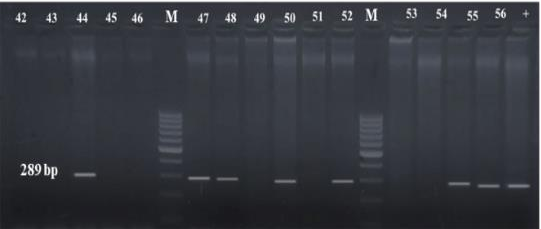

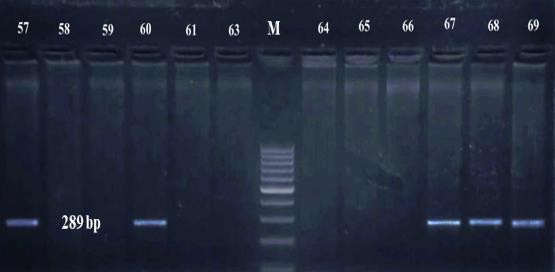

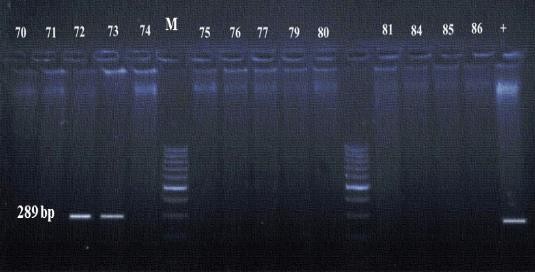

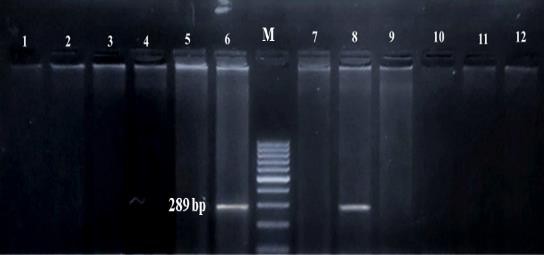

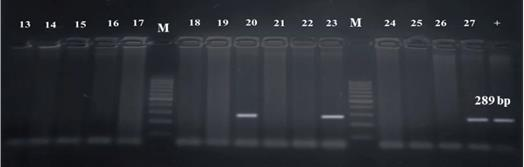


**Figure X1.** **PCR-based detection of *Lr*10 with an amplified product of 289bp on 4% MetaPhor agarose.**

(M- 100 bp ladder used, 1- NP 4, 2- NP 100, 3- NP 111, 4- NP 12, 5- NP 52, 6- NP 165, 7- C 591, 8- NP 710, 9- NP 718, 10- NP 745, 11- NP 760, 12- NP 761, 13- KENPHAD, 14- HY 12, 15- NP 770, 16- HY 5, 17- HYB 11, 18- C 281, 19- C 286, 20- C 285, 21- LERMA ROJA, 22- CHHOTI LERMA, 23- PV 18, 24- SHARBATI SONORA, 25- LALBAHADUR, 26- GW 10, 27- D 134, 28- K 816, 29- J 1-7, 30- WL 711, 31- HS 1138-6-4, 32- UP 262, 33- WL 410, 34- HP 1102, 35- HUW 12, 36- IWP 72, 37- KSML 3, 38- UP 115, 39- AJANTA, 40- HW 517, 41- MLKS 11, 42- UP 2003, 43- WL 1562, 44- PBW 54, 45- RAJ 1482, 46- SAGARIKA, 47- UP 2121, 48- DL 153-2, 49- GW 120, 50- HD 2307, 51- HUW 213, 52- J 405, 53- TAWA 267, 54- WH 291, 55- K 7410, 56- BW 11, 57- K 8020, 58- PBW 120, 59- PBW 138, 60- UP 1109, 61- H 1977, 62- HS 240, 63- HP 1633, 64- HS2 95, 65- PBN 51, 66- DL 784-3, 67- PBW 299, 68- HP 1731, 69- K 8962, 70- DL 788-2, 71- DDK 1009, 72- HS 365, 73- HW 1085, 74- NW 1014, 75- SONAK, 76- HI 454, 77- KRL 19, 78- PBW 396, 79- K 9162, 80- HUW 510, 81- HW 2045, 82- K 7903, 83- MP 4010, 84- RAJ 4037, 85- WR 544, and 86- HI 1500)


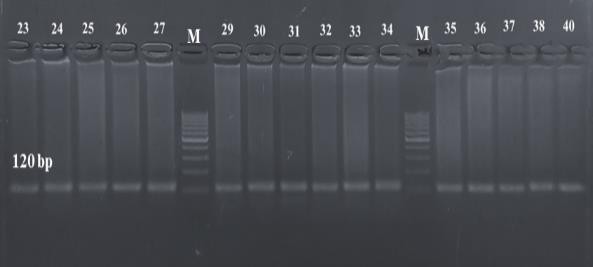

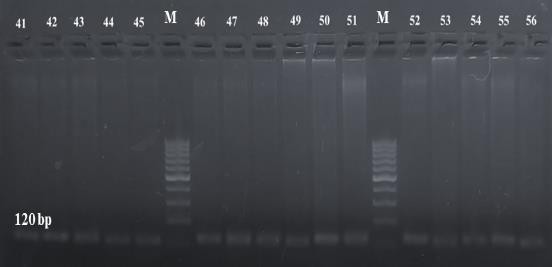

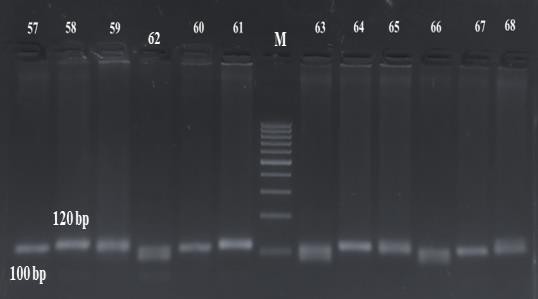

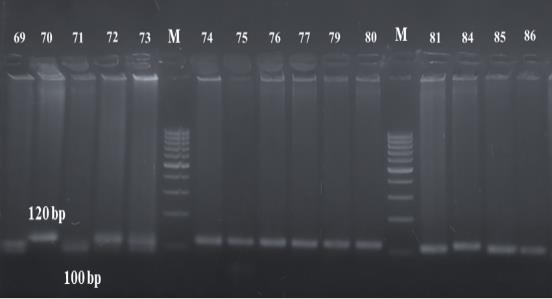

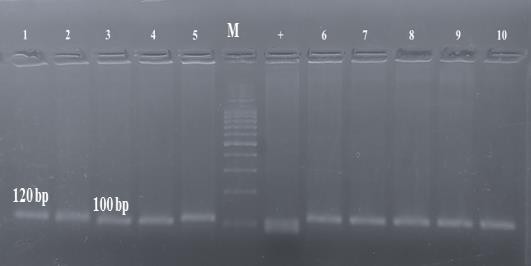

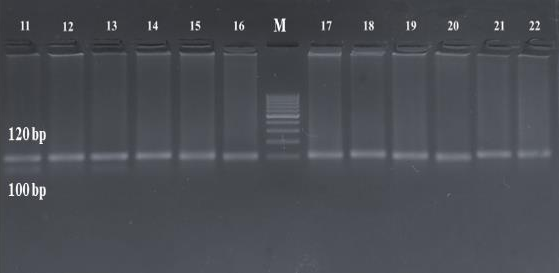


**Figure X2.** **The *Lr*24 (Xbarc71) primer amplified a band of 100 bp (presence) and 120 bp (absence) in all genotypes on 4% MetaPhor agarose.**

(M- 100 bp ladder used, 1- NP 4, 2- NP 100, 3- NP 111, 4- NP 12, 5- NP 52, 6- NP 165, 7- C 591, 8- NP 710, 9- NP 718, 10- NP 745, 11- NP 760, 12- NP 761, 13- KENPHAD, 14- HY 12, 15- NP 770, 16- HY 5, 17- HYB 11, 18- C 281, 19- C 286, 20- C 285, 21- LERMA ROJA, 22- CHHOTI LERMA, 23- PV 18, 24- SHARBATI SONORA, 25- LALBAHADUR, 26- GW 10, 27- D 134, 28- K 816, 29- J 1-7, 30- WL 711, 31- HS 1138-6-4, 32- UP 262, 33- WL 410, 34- HP 1102, 35- HUW 12, 36- IWP 72, 37- KSML 3, 38- UP 115, 39- AJANTA, 40- HW 517, 41- MLKS 11, 42- UP 2003, 43- WL 1562, 44- PBW 54, 45- RAJ 1482, 46- SAGARIKA, 47- UP 2121, 48- DL 153-2, 49- GW 120, 50- HD 2307, 51- HUW 213, 52- J 405, 53- TAWA 267, 54- WH 291, 55- K 7410, 56- BW 11, 57- K 8020, 58- PBW 120, 59- PBW 138, 60- UP 1109, 61- H 1977, 62- HS 240, 63- HP 1633, 64- HS2 95, 65- PBN 51, 66- DL 784-3, 67- PBW 299, 68- HP 1731, 69- K 8962, 70- DL 788-2, 71- DDK 1009, 72- HS 365, 73- HW 1085, 74- NW 1014, 75- SONAK, 76- HI 454, 77- KRL 19, 78- PBW 396, 79- K 9162, 80- HUW 510, 81- HW 2045, 82- K 7903, 83- MP 4010, 84- RAJ 4037, 85- WR 544, and 86- HI 1500)


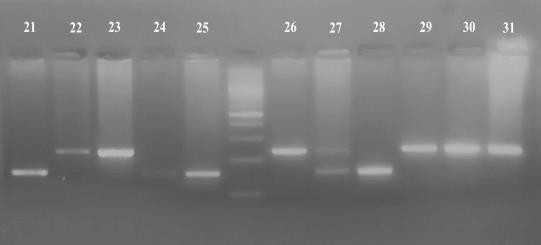

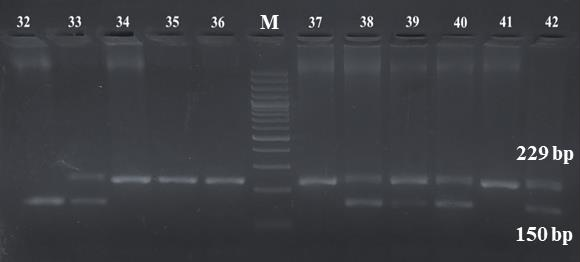

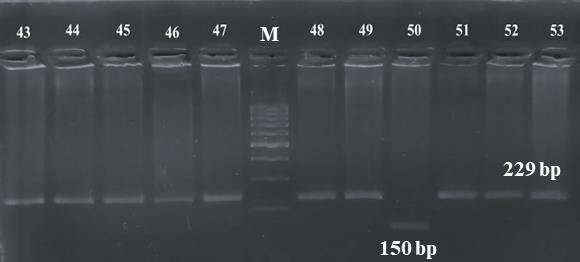

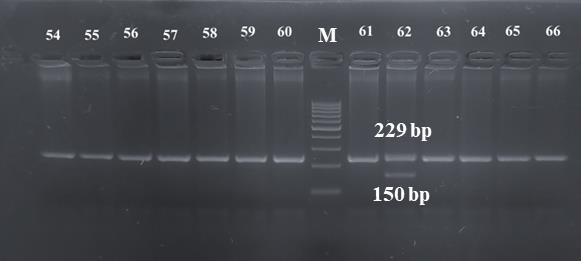

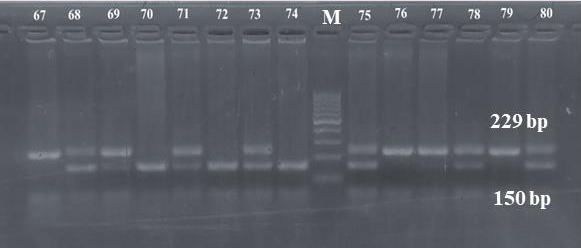

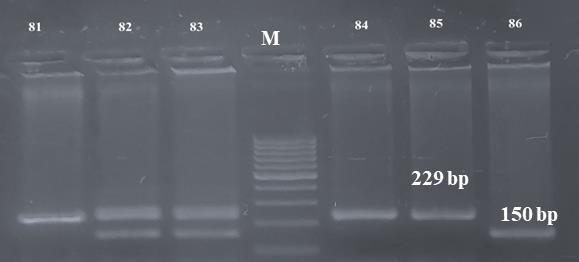

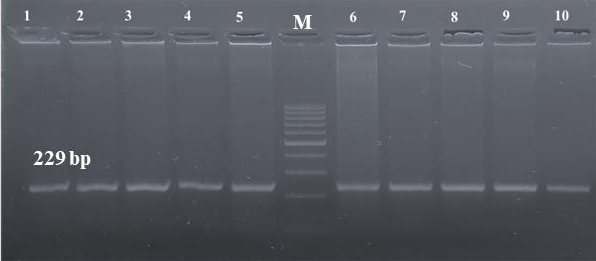

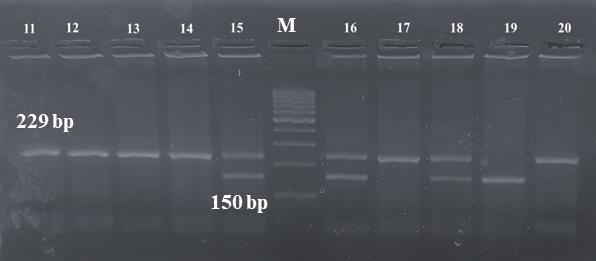


**Figure X3. The *Lr*34 (csLV34) primer amplified a band of 150 bp (presence) and 229 bp (absence) in all genotypes on 4% MetaPhor agarose.**

(M- 100 bp ladder used, 1- NP 4, 2- NP 100, 3- NP 111, 4- NP 12, 5- NP 52, 6- NP 165, 7- C 591, 8- NP 710, 9- NP 718, 10- NP 745, 11- NP 760, 12- NP 761, 13- KENPHAD, 14- HY 12, 15- NP 770, 16- HY 5, 17- HYB 11, 18- C 281, 19- C 286, 20- C 285, 21- LERMA ROJA, 22- CHHOTI LERMA, 23- PV 18, 24- SHARBATI SONORA, 25- LALBAHADUR, 26- GW 10, 27- D 134, 28- K 816, 29- J 1-7, 30- WL 711, 31- HS 1138-6-4, 32- UP 262, 33- WL 410, 34- HP 1102, 35- HUW 12, 36- IWP 72, 37- KSML 3, 38- UP 115, 39- AJANTA, 40- HW 517, 41- MLKS 11, 42- UP 2003, 43- WL 1562, 44- PBW 54, 45- RAJ 1482, 46- SAGARIKA, 47- UP 2121, 48- DL 153-2, 49- GW 120, 50- HD 2307, 51- HUW 213, 52- J 405, 53- TAWA 267, 54- WH 291, 55- K 7410, 56- BW 11, 57- K 8020, 58- PBW 120, 59- PBW 138, 60- UP 1109, 61- H 1977, 62- HS 240, 63- HP 1633, 64- HS2 95, 65- PBN 51, 66- DL 784-3, 67- PBW 299, 68- HP 1731, 69- K 8962, 70- DL 788-2, 71- DDK 1009, 72- HS 365, 73- HW 1085, 74- NW 1014, 75- SONAK, 76- HI 454, 77- KRL 19, 78- PBW 396, 79- K 9162, 80- HUW 510, 81- HW 2045, 82- K 7903, 83- MP 4010, 84- RAJ 4037, 85- WR 544, and 86- HI 1500)
